# Loss of Stemness, EMT, and Supernumerary Tooth Formation in *Cebpb*^−/−^*Runx2*^+/−^ Murine Incisors

**DOI:** 10.1038/s41598-018-23515-y

**Published:** 2018-03-26

**Authors:** Kazuyuki Saito, Katsu Takahashi, Boyen Huang, Masakazu Asahara, Honoka Kiso, Yumiko Togo, Hiroko Tsukamoto, Sayaka Mishima, Masaki Nagata, Machiko Iida, Yoshihito Tokita, Masato Asai, Akira Shimizu, Toshihisa Komori, Hidemitsu Harada, Mary MacDougall, Manabu Sugai, Kazuhisa Bessho

**Affiliations:** 1 Department of Oral and Maxillofacial Surgery, Graduate School of Medicine, Kyoto University, Kyoto, Japan; 2 School of Dentistry and Health Sciences, Faculty of Science, Charles Sturt University, Leeds Parade Orange, NSW 2800 Australia; 3 Division of Liberal Arts and Sciences, Aichi Gakuin University, Aichi, Japan; 4 Department of Oral and Maxillofacial Surgery Niigata University Graduate School of Medical and Dental Sciences, Niigata, Japan; 5 Department of Perinatology, Institute for Developmental Research, Aichi Human Service Center, Kasugai, Aichi Japan; 6 Department of Experimental Therapeutics, Institute for Advancement of Clinical and Translational Science, Kyoto University Hospital, Kyoto, Japan; 7 Department of Cell Biology, Unit of Basic Medical Sciences, Nagasaki University Graduate School of Biomedical Sciences, Nagasaki, Japan; 8 The Advanced Oral Health Science Research Center, Iwate Medical University, Iwate, Japan; 9 Facultyl of Dentistry, University of British Columbia, Vancouver, Canada; 10 Department of Molecular Genetics, Division of Medicine, Faculty of Medical Sciences, University of Fukui, Fukui, Japan

## Abstract

Adult *Cebpb* KO mice incisors present amelogenin-positive epithelium pearls, enamel and dentin allopathic hyperplasia, fewer *Sox2*-positive cells in labial cervical loop epitheliums, and reduced *Sox2* expression in enamel epithelial stem cells. Thus, *Cebpb* acts upstream of *Sox2* to regulate stemness. In this study, *Cebpb* KO mice demonstrated cementum-like hard tissue in dental pulp, loss of polarity by ameloblasts, enamel matrix in ameloblastic layer, and increased expression of epithelial-mesenchymal transition (EMT) markers in a *Cebpb* knockdown mouse enamel epithelial stem cell line. *Runx2* knockdown in the cell line presented a similar expression pattern. Therefore, the EMT enabled disengaged odontogenic epithelial stem cells to develop supernumerary teeth. *Cebpb* and *Runx2* knockdown in the cell line revealed higher *Biglycan* and *Decorin* expression, and *Decorin*-positive staining in the periapical region, indicating their involvement in supernumerary tooth formation. *Cebpb* and *Runx2* acted synergistically and played an important role in the formation of supernumerary teeth in adult incisors.

## Introduction

Alterations in tooth development have enabled us to study a variety of dental anomalies, with supernumerary teeth (extra teeth) and tooth agenesis (missing teeth) being among the most common ones in humans. That said, numerous mouse mutant models have provided insights into the formation of supernumerary teeth^1–13^. We previously showed that inhibition of apoptosis can lead to the successive development of rudimentary maxillary incisors in uterine sensitization-associated gene-1 (*Usag-1*) null mice^5,14,15^. We also demonstrated that CCAAT/enhancer binding protein (C/EBP) beta (*Cebpb*) deficiency was related to the formation of supernumerary teeth. A total of 66.7% of *Cebpb*^−/−^ 12-month-old animals presented supernumerary teeth and/or odontomas^1^. We found mineralized tissue formation typical of supernumerary teeth, around the labial cervical loop epithelium in 3-month-old adult mice, whereas no phenotypic changes were observed during embryonic tooth development. However, the mechanism of supernumerary tooth formation around the labial cervical loop epithelium in adulthood remains unclear.

We recently found interesting phenotypes in *Usag-1*/runt-related transcription factor 2 (*Runx2*) double knockout mice: the prevalence of supernumerary teeth was lower than that of *Usag-1* null mice and the frequency of molar lingual buds were lower than that of *Runx2* null mice. Hence, we suggested that *Runx2* and *Usag-1* act in an antagonistic manner in supernumerary tooth formation^16^, with *Runx2* negatively regulating the process.

Endogenous *Cebpb* is expressed in ameloblasts and odontoblasts of 6-month-old wild-type mouse incisors^17^. *Runx2* is expressed in the dental epithelium and/or mesenchyme of both the incisors and molars, and exhibits distinct temporospatial patterns^18–21^. Mutations in *Runx2* are responsible for inherited cleidocranial dysplasia (CCD), an autosomal-dominant disorder characterized by elevated supernumerary tooth formation^22,23^. Interestingly, both heterozygous and null *Runx2* mice possess a lingual epithelial bud, which represents a putative successional tooth associated with the upper molars and incisors; however, supernumerary tooth formation has never been observed in *Runx2*^+/−^ mice^24,25^. The dental abnormalities in CCD suggest that *Runx2* plays an important role during dental formation. Although *Runx2* has been considered a determinant of CCD, some CCD patients do not present *Runx2* mutations. We demonstrated previously in mice that prospective signs of CCD could be associated with *Cebpb* deficiency^26^. These could provide an additional etiological factor of CCD. *Cebpb*^−/−^*Runx2*^+/−^ mice have shown more severe dwarfism than their *Cebpb*^−/−^ littermates during the perinatal period and remained smaller even 12 weeks after birth. Thus, *Cebpβ* and *Runx2* collaboratively control skeletal growth and osteoarthritis during development^27^, however their combined role in tooth morphogenesis remains unknown.

Given the above evidence, we hypothesized that *Cebpb* and *Runx2* acted synergistically and played an important role in tooth morphogenesis and the formation of supernumerary teeth around the labial cervical loop epithelium in adult incisors. To test our hypothesis and study the *in vivo* relationship between *Cebpb* and *Runx2* we established double knockout mice.

## Results

### *Cebpb* is associated with the maintenance of stemness in odontogenic epithelial stem cells and the labial cervical loop epithelium of adult maxillary incisors

**Figure 1:**
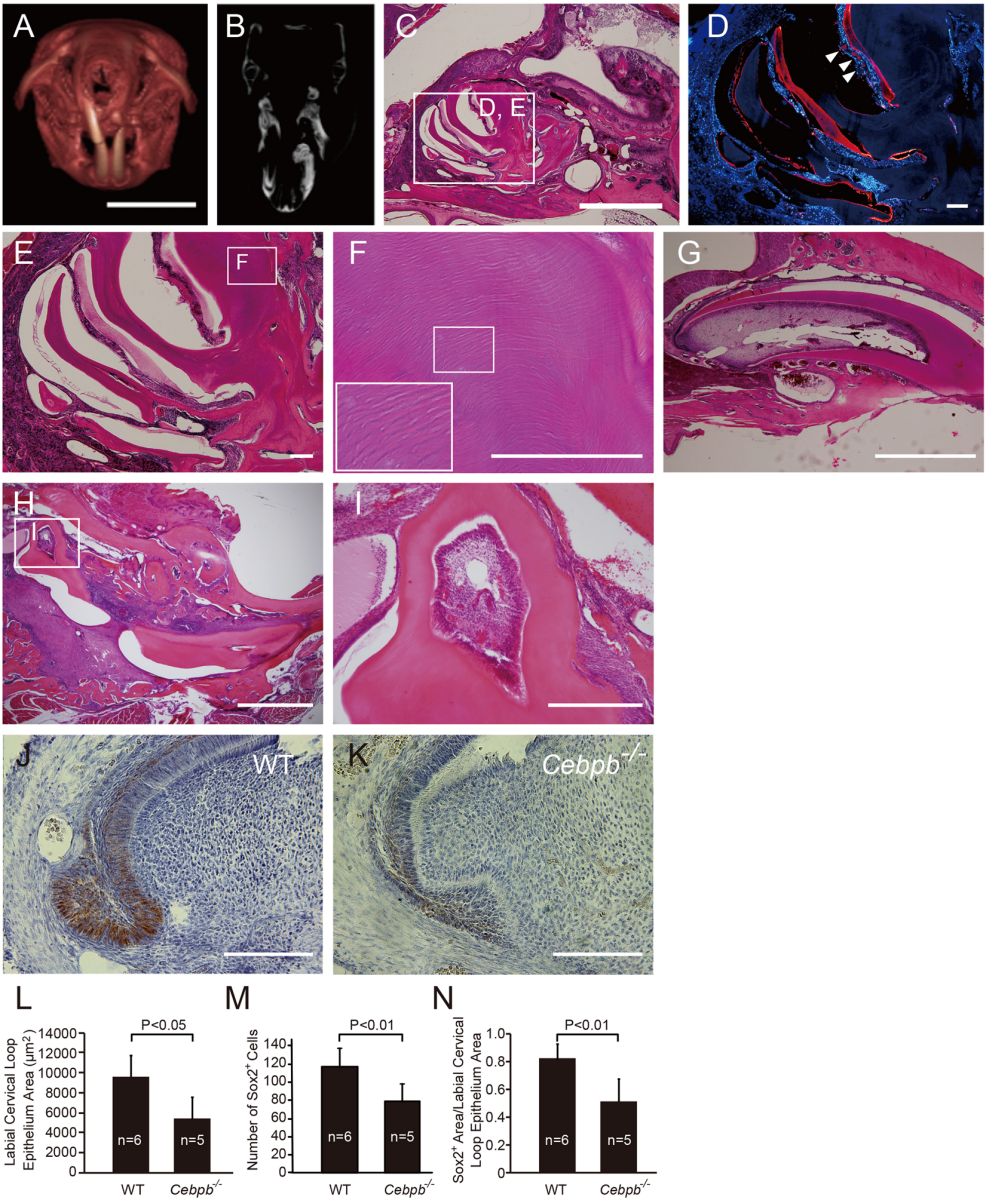
Phenotypes of incisors in *Cebpb*^−/−^ mice maintained in a 129 Sv background. (**A**) Three-dimensional image of the skull. The left incisor is short. Scale bar: 10 mm. (**B**) Micro-computed tomography (CT) axial section image at the level of the maxillary incisor. Left incisors are deformed. (**C**) Sagittal hematoxylin-eosin (H&E)-stained section of the left upper incisor. Ectopic marked growth of dentin and enamel is observed in the apical region of the left incisor. Scale bar: 1 mm, 40×. (**D**) Amelogenin immunostaining (red) and Hoechst nuclear staining (blue). Amelogenin is observed in the hypocalcification site. Mature enamel was removed from this decalcified section leaving a clear enamel space (ES). Arrowheads indicate epithelial pearls. Scale bar: 100 μm, 100×. (**E**) Many dental tubules can be seen in dentin, which is also more abundant. Derangement of ameloblasts are observed near enamel matrix. Scale bar: 100 μm, 200×. (**F**) Magnified image of dental tubules in dentin. Scale bar:200 μm. 400× Inset: 1000×. (**G**) Nearly normal development of the right incisor. Ameloblasts, enamel-producing cells, are arranged in a single-cell row. Odontoblasts, dentin-producing cells, line the pulp cavity. Increased dentin is observed in both the labial and lingual side. Miniaturization of the labial cervical loop epithelium is seen. Scale bar: 1 mm, 40×. (**H**) Sagittal H&E-stained section of the left mandibular incisor of a 10-month-old mouse. Scale bar: 2 mm, 20×. (**I**) The boxed region in H. A mature tooth structure with pulp enclosed with dentin in a different direction is observed in a periapical region of a mandibular incisor. Scale bar: 500 μm, 100×. (**J**) SRY (sex determining region Y)-box 2 (*Sox2*)*-*Positive cells in a maxillary incisor labial cervical loop epithelium of 1-month-old wild-type (WT) mice. Scale bar: 100 μm, 400×. (**K**) Sox2-Positive cells in a maxillary incisor labial cervical loop epithelium of 19-month-old *Cebpb*^−/−^ mice. Scale bar: 100 μm, 400×. (**L**) Comparison between WT and *Cebpb*^−/−^ mice. Y-axis indicates the labial cervical loop epithelium area; X-axis indicates genotypes. (**M**) Comparison between WT and *Cebpb*^−/−^ mice. Y-axis indicates number of Sox2-positive cells in the labial cervical loop epithelium; the X-axis indicates genotypes. (**N**) Comparison between WT and *Cebpb*^−/−^ mice. Y-axis indicates Sox2-positive area in the labial cervical loop epithetium area; X-axis indicates genotypes.

To investigate the mechanisms underlying mineralized tissue formation around the labial cervical loop epithelium, such as those seen in supernumerary tooth structures, we first performed a detailed histological evaluation of adult *Cebpb*^−/−^ mice (129 Sv background) (Fig. 1A–C). We observed numerous amelogenin-positive epithelium pearls near the periapical tissue (Fig. 1D), enamel and dentin ectopic hyperplasia in the periapical tissue (Fig. 1B–E,H), as well as miniaturization of the labial cervical loop epithelium that is specialized epithelial structure for stem cell niche (Fig. 1G,K–N). Based on these results, we concluded that odontogenic epithelial stem cells (OESCs) within the labial cervical loop epithelium lost their stemness in *Cebpb*^−/−^ mice. Consequently, these OESCs differentiated into ameloblasts and secrete enamel matrix protein and enamel ectopically. Ectopic enamel formation was observed mainly in the maxilla, and was also observed in the mandible with low frequency (in a 129 Sv background) (Fig. 1H,I)^1^. We therefore investigated SRY (sex determining region Y)-box 2 (Sox2) expression in the labial cervical loop epithelium of maxillary incisors in *Cebpb*^−/−^ mice. Sox2 localized to the inner and outer enamel epithelium, particularly the transit-amplifying zone, as well as the stellate reticulum (Fig. 1J), confirming a previous report^28^. There were significantly fewer Sox2-positive cells in the labial cervical loop epithelium of adult *Cebpb*^−/−^ mouse incisors than in wild-type animals (Fig. 1J–N). These findings suggest that *Cebpb* maintains Sox2*-*positive OESCs in the labial cervical loop epithelium during postnatal life.

### Abrogation of *Cebpb* and *Runx2* works synergistically in supernumerary tooth formation around the labial cervical loop epithelium in adult *Cebpb*^−/−^*Runx2*^+/−^ mice

**Figure 2:**
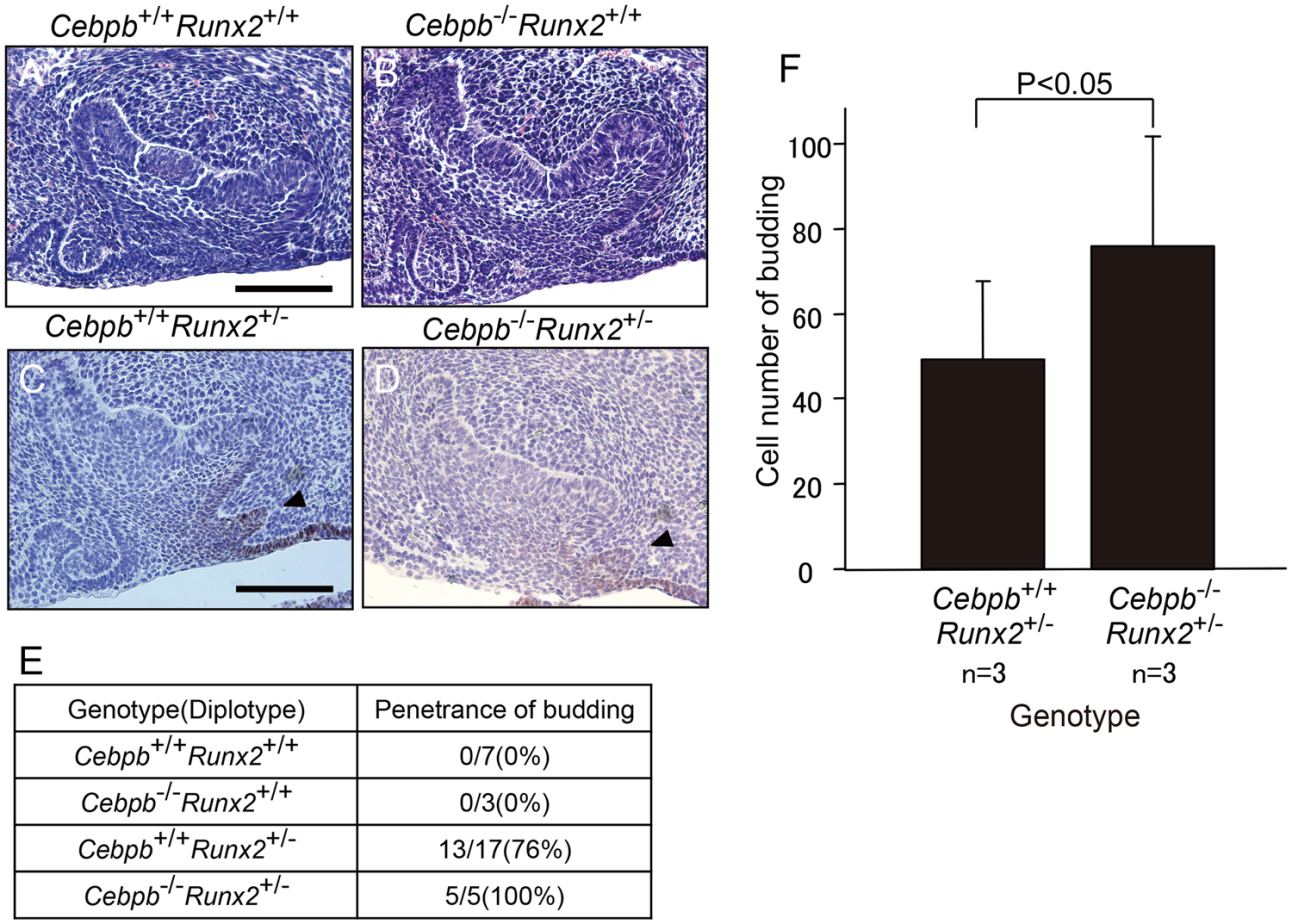
Phenotype of maxillary incisor germ in the F_2_ generation at embryonic day 15. (**A**,**B**) Incisor germs of *Cebpb*^+/+^*Runx2*^+/+^ and *Cebpb*^−/−^*Runx2*^+/+^ mice. Scale bar: 100 μm, 400×. Left indicates the labial side. (**C**,**D**) Immunostaining for SRY (sex determining region Y)-box 2 (*Sox2*) in incisor germs of *Cebpb*^+/+^*Runx2*^+/−^ and *Cebpb*^−/−^*Runx2*^+/−^ mice. Scale bar: 100 μm, 400×. Left indicates labial side. *Runx2*^+/−^ tooth germ displays lingual budding(arrowhead). (**E**) Penetrance of budding according to genotype. (**F**) Comparison between *Cebpb*^+/+^*Runx2*^+/−^ and *Cebpb*^−/−^*Runx2*^+/−^ mice lingual budding. The Y-axis on the left indicates the cell number of buddings in the maximal area of all sections; the X-axis on the bottom indicates genotypes.

**Figure 3:**
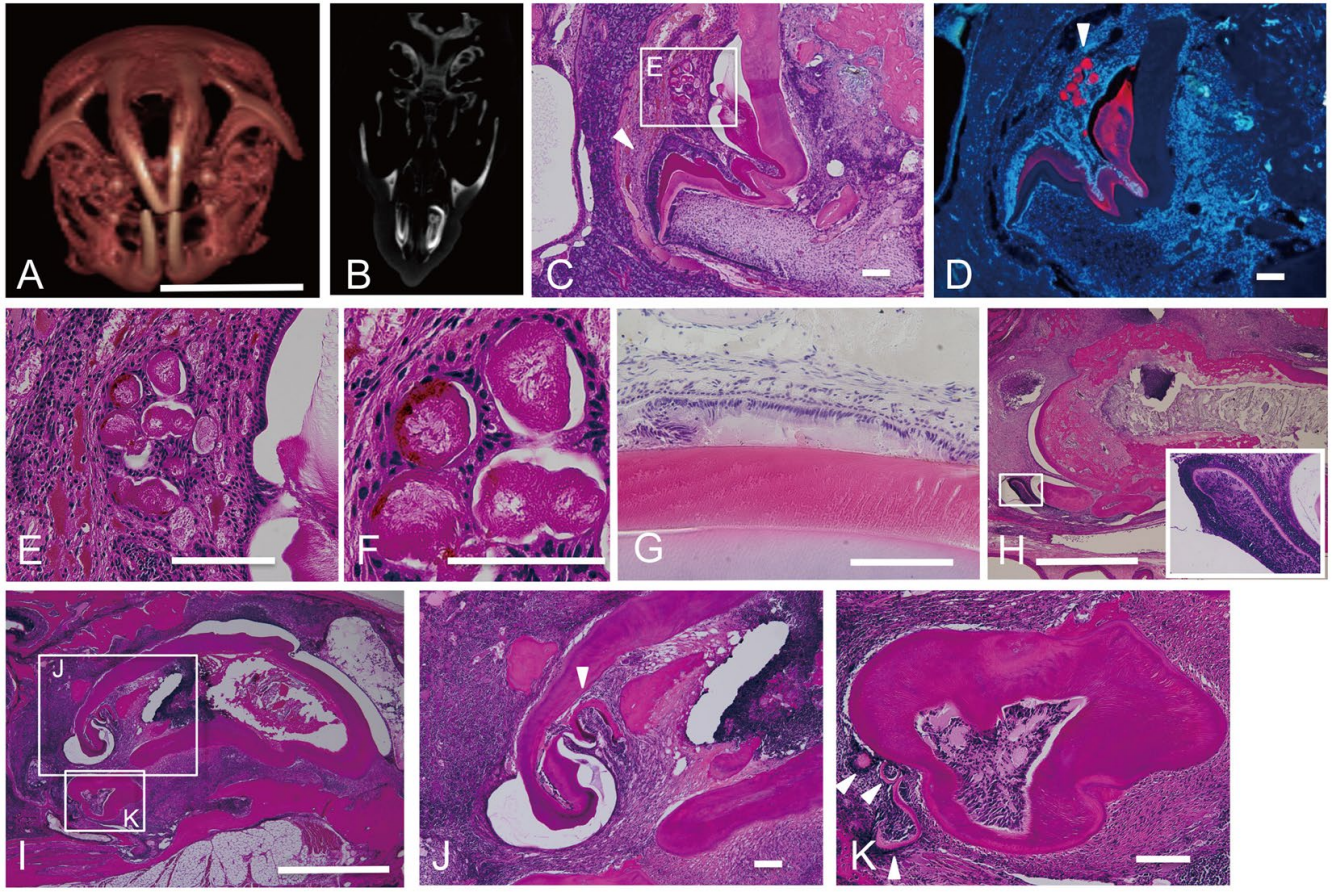
Phenotypes of maxillary incisors in the F_2_ generation of 3-month-old *Cebpb*^−/−^*Runx2*^+/−^ mice. All sections were decalcified. (**A**–**G**) Correspond to the same mouse, H corresponds to another animal, and (**I**–**K**) correspond to the other animal. (**A**) Three-dimensional image of the skull. Scale bar: 10 mm. (**B**) Micro-computed tomography (CT) axial image at the level of the maxillary incisor. (**C**) Sagittal hematoxylin-eosin (H&E)-stained section of the upper incisor. A box E indicates epithelial pearls. An arrowhead indicates a developing tooth. Developing teeth are characterized by odontoblasts, dentin, ameloblasts derangement, and surface roughness of the enamel matrix (hypocalcification site). Scale bar: 100 μm, 100×. (**D**) Amelogenin immunostaining (red) and Hoechst nuclear staining (blue). An arrowhead indicates epithelial pearls. Scale bar: 100 μm, 100×. (**E**) High-magnification image of epithelial pearls. Scale bar: 100 μm, 400×. (**F**) High-magnification image of epithelial pearls. Scale bar: 50 μm, 1000×. (**G**) Ameloblasts derangement and surface roughness of the enamel matrix are visible in an opposite incisor. Scale bar: 100 μm, 400×. (**H**) Two mature teeth and a developing tooth are visible. Scale bar: 1 mm, 40×. Inset: Higher magnification image of a developing tooth showing odontoblasts, ameloblasts, and dentin 400×. (**I**) Developing and mature teeth are visible. Scale bar: 1 mm, 40×. (**J**) A multipartite mature tooth (arrowhead) is observed in the dental pulp. Scale bar: 100 μm, 100×. (**K**) Many developing teeth (arrowheads) are visible near an extra mature tooth. Scale bar: 100 μm, 200×.

We had previously observed mineralized tissue, akin to that of supernumerary tooth structures, around the labial cervical loop epithelium in adult *Cebpb*^−/−^ mice^1^. Although *Runx2*^−/−^ mice die shortly after birth due to the absence of bone formation^22^, some *Runx2*^+/−^ mice, showed lingual bud formation in the embryonic stage. Therefore, to determine whether *Cebpb* and *Runx2* acted synergistically and played an important role in supernumerary tooth formation, we performed a series of histological studies of maxillary incisor formation in *Cebpb*^+/+^*Runx2*^+/+^, *Cebpb*^−/−^*Runx2*^+/+^, *Cebpb*^+/+^*Runx2*^+/−^, and *Cebpb*^−/−^*Runx2*^+/−^ mice (Fig. 2A–D). We first investigated the synergistic action of *Cebpb* and *Runx2* during lingual bud formation. We previously reported that *Usag-1* and *Runx2* acted in an antagonistic manner in lingual bud formation at embryonic day 15 (E15)^16^. Here, the *Cebpb*^+/+^*Runx2*^+/−^ (F_2_ background) maxillary incisor germ displayed lingual budding at E15 (Fig. 2C); however this genotype was smaller incidence rate of budding (Fig. 2E) and was significantly fewer cell number of budding than *Cebpb*^−/−^*Runx2*^+/−^ (Fig. 2F). These results support the hypothesis that *Cebpb* and *Runx2* act synergistically in OESCs during embryonic development. In contrast, the lingual bud was highly enriched with *Sox2*-positive OESCs. At present, we could not find out how OESCs within the lingual bud lost their stemness during the embryonic stage. Next, we analyzed whether *Cebpb* and *Runx2* acted synergistically during supernumerary tooth formation around the labial cervical loop epithelium in adult mice (Fig. 3). Five out of eight (62.5%) adult *Cebpb*^−/−^*Runx2*^+/+^ (F_2_ background) mice presented mineralized tissue around the labial cervical loop epithelium, akin to that of supernumerary tooth structures, roughly reflecting the value reported in *Cebpb*^−/−^ (129 Sv background) mice (66.7%)^1^ (Table 1). In severe cases, no labial cervical loop epithelium structures were seen in the aberrant incisors. These results indicate that there is no phenotypic difference between F_2_ and 129 Sv mouse background. Four (33%) out of twelve *Cebpb*^−/−^*Runx2*^+/−^ 3-month-old (F_2_ background) mice presented aberrant maxillary incisors, with developing or mature ectopic supernumerary teeth in the periapical tissue and dental pulp (Table 1, Fig. 3A–D,H–K). The incidence of short incisors was higher in *Cebpb*^−/−^ mice (129 Sv or F_2_ background) than in *Cebpb*^−/−^*Runx2*^+/−^ mice (F_2_ background), whereas the incidence of supernumerary teeth showed the opposite trend (Table 1). These data confirm the synergistic action of *Cebpb* and *Runx2* in supernumerary tooth formation around the labial cervical loop epithelium during postnatal life. Moreover, this is the first report on supernumerary tooth formation in *Cebpb*^−/−^*Runx2*^+/−^ mice.

**Table 1:** Phenotype of *Cebpb* and *Runx2* genetically modified mice. (F_2_:129 Sv/C57BL/6) with aberrant incisors at 3 months after birth. The table shows penetrance, location, and characteristics of aberrant incisors.

	*Cebpβ*+/+*Runx2*+/+ (F2)	*Cebpβ*+/+*Runx2*+/−(F2)	*Cebpβ*−/−*Runx2*+/+ (F2)	*Cebpβ*−/−*Runx2*+/− (F2)
Penetrance	0/4 (0%)	0/9 (0%)	5/8 (62.5%)	4/12 (33%)
Location of aberrant incisor				
Maxilla	0(0%)	0(0%)	5(100%)	4(100%)
Mandible	0(0%)	0(0%)	0(0%)	0(0%)
Unilateral	0(0%)	0(0%)	1(20%)	4(100%)
Bilateral	0(0%)	0(0%)	4(80%)	0(0%)
Characteristics of aberrant incisors				
Short incisor	0(0%)	0(0%)	3(60%)	0(0%)
With supernumerary teeth	0(0%)	0(0%)	2(40%)	4(100%)

### Reduction of *Cebpb* and *Runx2* expression contributes to epithelial-mesenchymal transition (EMT) in odontogenic epithelial cells of the maxillary incisor

**Figure 4:**
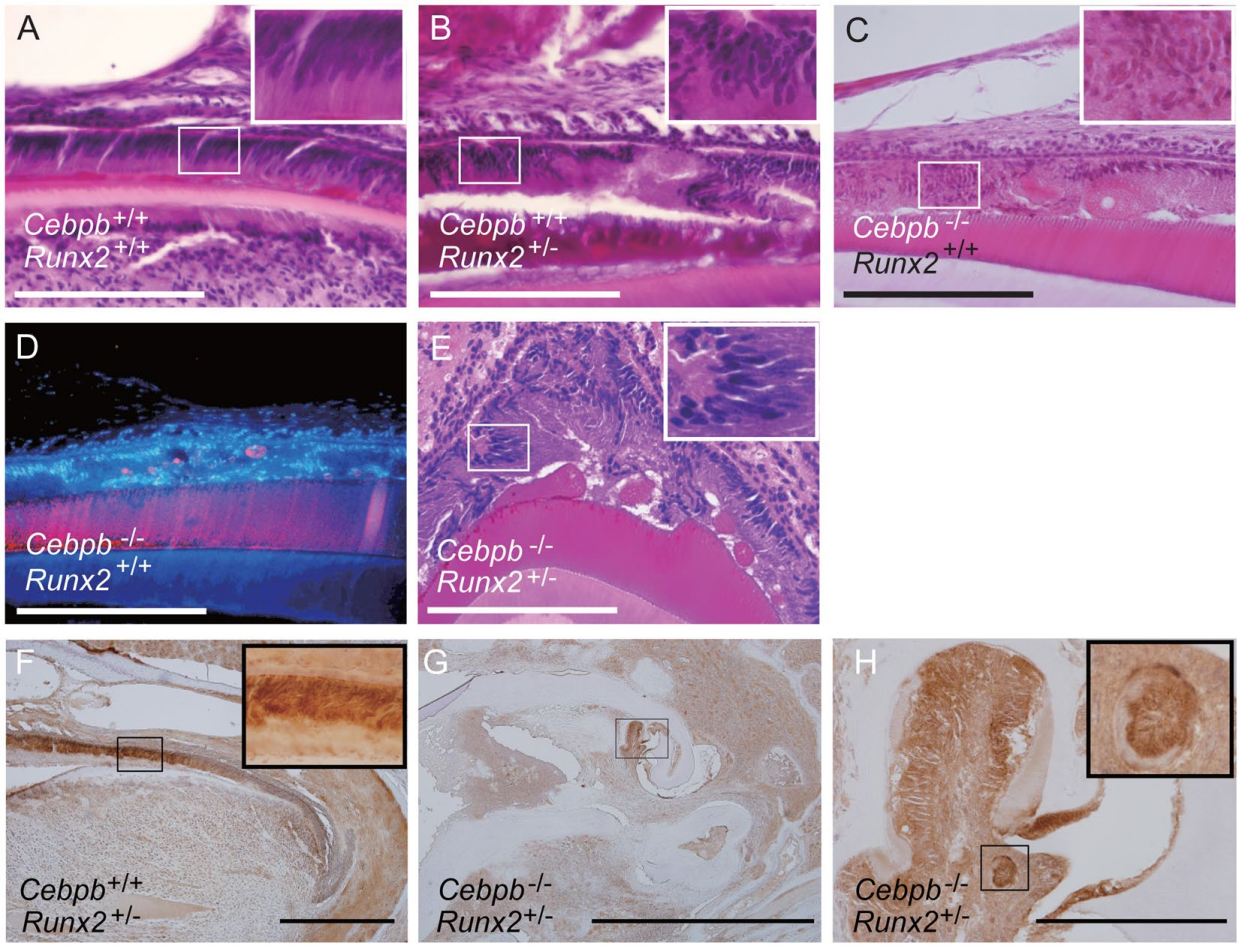
Analysis of differentiated ameloblasts in maxillary incisors of 3-month-old mice (F_2_ generation). All sections were decalcified. These insets in (**A**–**C**,**E**) are 1000×. (**F**–**H**) N-cadherin immunostaining. All scale bars: 200 μm, 400×. (**A**) Highly polarized secretory ameloblasts are visible in wild-type mice. (**B**) Differentiated ameloblasts are seen to have lost their cell polarity in *Cebpb*^+/+^*Runx2*^+/−^ mice. (**C**) Non-polarized differentiated ameloblasts and surface roughness of the enamel matrix are visible in *Cebpb*^−/−^*Runx2*^+/+^ mice. (**D**) Amelogenin immunostaining (red) and Hoechst nuclear staining (blue). Free blobs containing amelogenin are observed in *Cebpb*^−/−^*Runx2*^+/+^ mice. (**E**) Derangement of ameloblasts and surface roughness of the enamel matrix. in *Cebpb*^−/−^ *Runx2*^+/−^ mice. (**F**) Positive staining in ameloblasts containing non-polarized differentiated ameloblasts and labial cervical loop epithelium in *Cebpb*^+/+^*Runx2*^+/−^ mice incisor. Scale bar: 500μm, 100×. The inset: High-magnification image of non-polarized differentiated ameloblasts. 1000×. (**G**) Ectopic enamel formation and cementum-like hard tissue within the dental pulp in the periapical region of *Cebpb*^−/−^ *Runx2*^+/−^ mice incisor. Scale bar: 2 mm, 40×. (**H**) Positive staining of the cell component surrounding ectopic enamel formation and cementum-like hard tissue are visible. Scale bar: 200 μm, 400×. The inset: High-magnification image of the cementum-like hard tissue 1000×.

With an aim to examine the effect of *Runx2*^+/−^ on postnatal supernumerary tooth formation, we performed a detailed histological evaluation around the labial cervical loop epithelium. Compared to wild-type mice (Fig. 4A), differentiated ameloblasts in the maxillary incisor were deranged and lost their cell polarity in adult *Cebpb*^+/+^*Runx2*^+/−^ animals (F_2_ background) (Fig. 4B). Furthermore, loss of cell polarity and surface roughness of the enamel matrix were visible also in *Cebpb*^−/−^*Runx2*^+/+^ and *Cebpb*^−/−^*Runx2*^+/−^ mice (Fig. 4C–E). Concomitant to the EMT process is the disappearance of the apical-basal polarity of epithelial cells^29^. Therefore, we performed immunostaining for the EMT marker-N cadherin-in the maxillary incisors of 3-month-old *Cebpb*^+/+^*Runx2*^+/−^ and *Cebpb*^−/−^*Runx2*^+/−^ mice. Non-polarized differentiated ameloblasts and the cell component surrounding ectopic enamel formation and cementum-like hard tissue within the dental pulp in the periapical region were positive for presence of N-cadherin (Fig. 4F–H). Thus, based on these morphological changes, we suggest that *Cebpb* and *Runx2* knockdown contributed to EMT in odontogenic epithelial cells of the maxillary incisor. Taken together, supernumerary tooth formation around the labial cervical loop epithelium of adult maxillary incisors is dependent on both *Cebpb* knockdown-induced loss of stemness in OESCs and EMT of odontogenic epithelial cells in *Runx2*^+/−^ and/or *Cebpb*^−/−^ mice.

### Reduced *Cebpb* mRNA expression leads to a decrease of *Sox2*, *Runx2* knockdown inhibits adhesion molecules and EMT in OESCs

**Figure 5:**
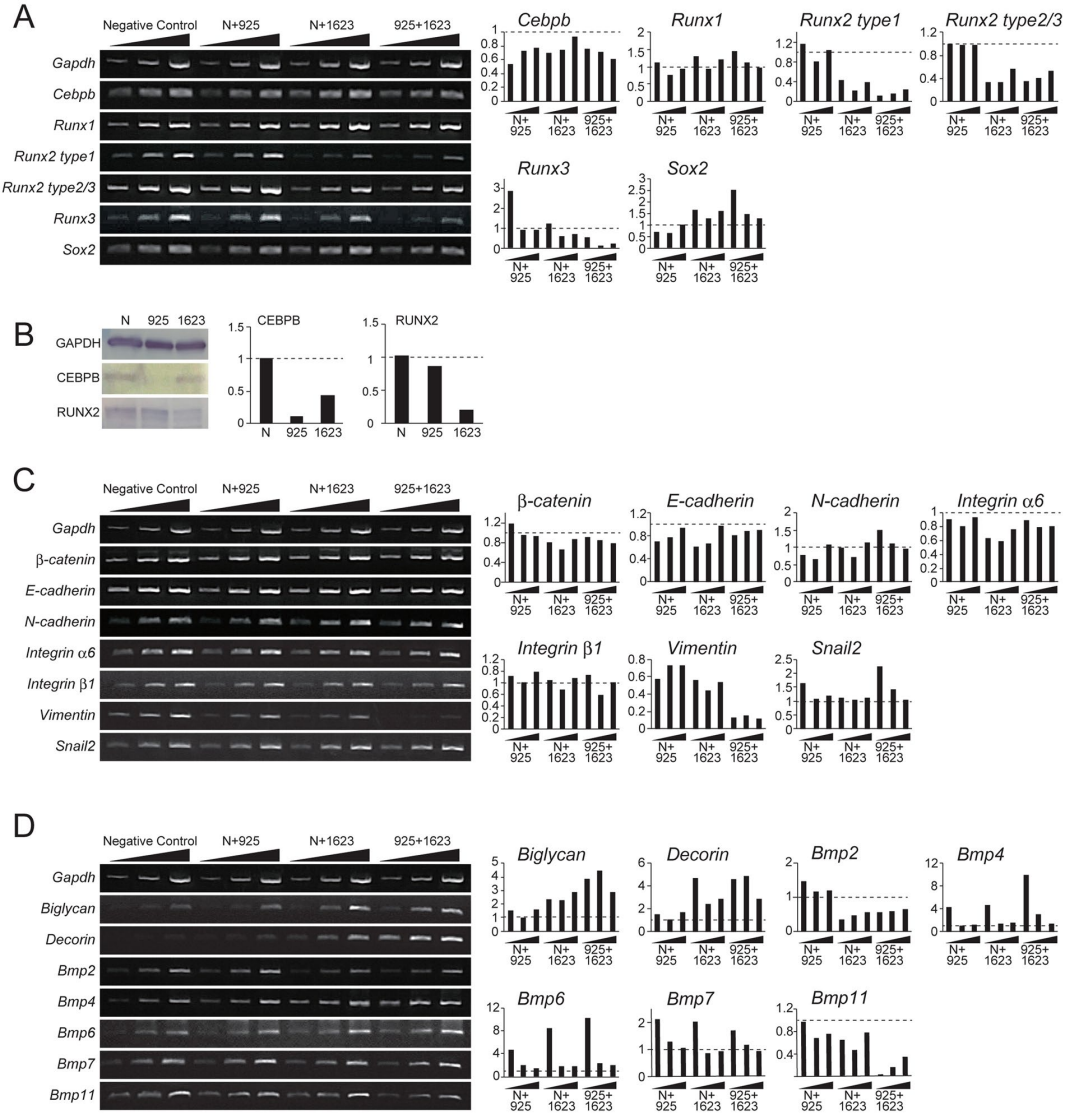
Semi-quantitative reverse transcription polymerase chain reaction (RT-PCR) and Western blotting results in mHAT9d cells 48 h after transfection with *Cebpb* and *Runx2 type1* stealth siRNA. (**A**) Changes in the expression of *Cebpb*, members of the *Runx* family, and SRY (sex determining region Y)-box 2 (*Sox2*) in mHAT9d cells 48 h after transfection with *Cebpb* and *Runx2 type1* stealth siRNA. *Cebpb* knockdown (N(negative control) + 925) shows slightly reduced expression of *Sox2*. *Runx2* knockdown (N + 1623) shows increased expression of *Sox2* and *Runx1*, but decreased expression of other *Runx2* isoforms and *Runx3*. Concomitant *Cebpb* and *Runx2* type1 siRNA knockdown (925 + 1623) was also performed. (**B**) Western blotting results. N indicates negative control stealth siRNA, 925 and 1623 indicate *Cebpb* and *Runx2* type1 siRNA, respectively (final concentration 10 nM). (**C**) Changes in the expression of adhesion molecules, epithelial-mesenchymal transition (EMT) markers, and mesenchymal markers in mHAT9d cells 48 h after transfection with *Cebpb* and *Runx2* type1 stealth siRNA. *Cebpb* knockdown (N(Negative control) + 925) caused increased expression of EMT markers snail family zinc finger 2 (*Snai2*) and decreased expression of the mesenchymal marker vimentin (*Vim*) and the epithelial marker E-cadherin (*Cdh1*). *Runx2* type1 knockdown (N + 1623) caused decreased expression of adhesion molecules *Cdh1* and integrin alpha 6 (*Itga6*), decreased expression of *Vim*, and increased expression of *Snai2*. Concomitant *Cebpb* and *Runx2* type1 knockdown (925 + 1623) caused decreased expression of *Cdh1*, *Itga6 and VIm*, and increased expression of mesenchymal marker N-cadherin (*Cdh2*) and *Snai2*. (**D**) Changes in biglycan (*Bgn*), decorin (*Dcn*), bone morphogenic proteins (*Bmp*s) in mHAT9d cells 48 h after transfection with *Cebpb* and *Runx2* type1 stealth siRNA. Concomitant *Cebpb* and *Runx2* type1 siRNA knockdown (925 + 1623) shows a markedly increased expression of *Bgn* and *Dcn* (encoding two small leucine-rich peptidogycans), and increased expression of *Bmp4/6*.

**Figure 6:**
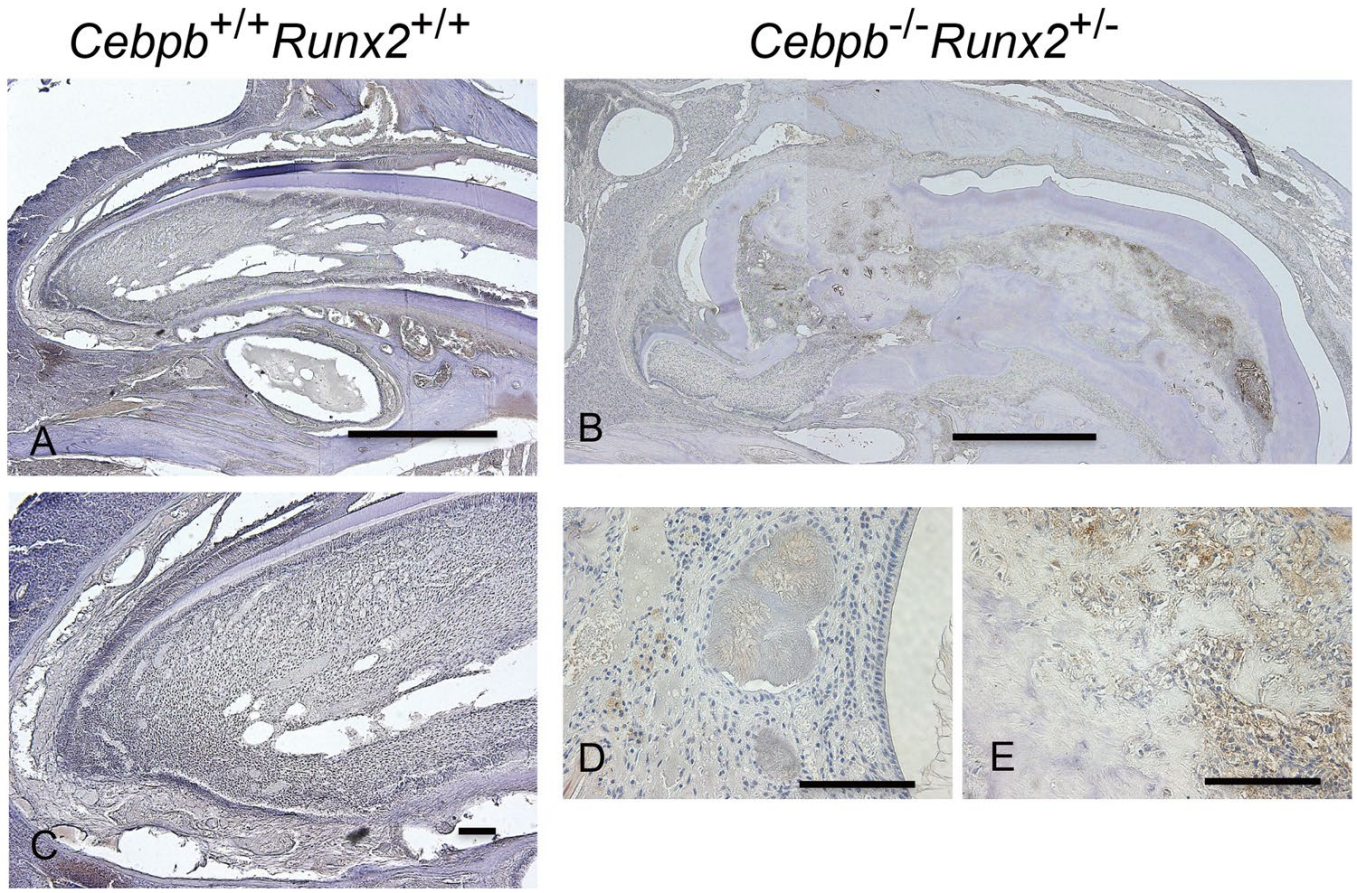
Decorin immunostaining in the incisors of 3-month-old *Cebpb*^−/−^*Runx2*^+/−^ mice (F_2_ generation). (**A**,**C**) Wild-type (WT)/WT mice Scale bar: 1 mm, 40×. (**B**,**D**,**E**) *Cebpb*^−/−^*Runx2*^+/−^ mice. (**B**) Positive staining of cells in the dental pulp, except for cementum-like hard tissue, and the extracellular matrix. Scale bar: 1 mm, 40×. (**D**) Positive staining in the enamel epithelial pearl and extracellular matrix near the supernumerary tooth in the periapical region. Scale bar: 100 μm, 400×. (**E**) Positive staining in the extracellular matrix surrounding the cementum-like hard tissue in the dental pulp. Scale bar: 100 μm, 400×.

To confirm the above-mentioned function of *Cebpb and Runx2* in odontogenic epithelial cells of the adult labial cervical loop epithelium, *in vitro* knockdown experiments were performed using mHAT9d cells. These cells are derived from the labial cervical loop epithelium of a mouse incisor and could undergo EMT^30^. Transfection efficiency was checked using a fluorescence-activated cell sorter (FACS) and was determined to be approximately 60% 24 h after addition of 1.5 µL Lipofectamine RNAiMAX and control stealth siRNA (Supplement Fig. 1). We used a semi-quantitative reverse transcription polymerase chain reaction (sqRT-PCR) to evaluate *Cebpb* and *Runx2* type1 knockdown by stealth siRNA in mHAT9d cells 48 h after transfection (Fig. 5). The specific stealth siRNA effectively abolished *Cebpb* and *Runx2* mRNA and protein levels (Fig. 5A,B). Furthermore, to confirm the specificity for the *Runx2* type1 knockdown, we also investigated the expression of *Runx1*, other *Runx2* isoforms, and *Runx3* mRNA. We found that expression of other *Runx2* isoforms and *Runx3* declined, whereas that of *Runx1* increased. Additionally, we showed that *Cebpb* acted upstream of *Sox2* to maintain stemness of mHAT9d cells, as indicated by reduced *Sox2* mRNA expression following *Cebpb* knockdown (Fig. 5A). However, expression of two ameloblast differentiation markers, amelogenin (*Amelx*) and ameloblastin (*Ambn*), could not be detected (Supplement Fig. 2). These results indicate that *Cebpb* alone is not sufficient for maintaining stemness and differential inhibition in mHAT9d cells *in vitro*. Next, we analyzed whether *Runx2* type1 knockdown showed EMT in mHAT9d cells. SqRT-PCR analysis of *Runx2* type1 knockdown by stealth siRNA demonstrated decreased mRNA expression of adhesion molecules (Fig. 5C). Expression of EMT markers snail family zinc finger 2 (*Snai2*) increased, as did that of the mesenchymal marker N-cadherin (*Cdh2*), whereas another mesenchymal marker, vimentin (*Vim*), and the epithelial marker E-cadherin declined. Similarly, *Cebpb* knockdown showed increased expression of *Snai2*, but a reduction of *Vim* and E-cadherin (Fig. 5C). Biglycan (*Bgn*), decorin (*Dcn*), and bone morphogenetic protein 4/6/7 (*Bmp4/6/7*) mRNA expression was seen to increase in *Runx2* type1 knockdown, and even more in *Cebpb* and *Runx2* type1 knockdown (Fig. 5D). Additionally, bone morphogenetic protein (*Bmp*2/11) mRNA expression decreased in *Runx2* type 1 knockdown and *Cebpb* and *Runx2* type 1 knockdown (Fig. 5D). Because *Dcn*, encoding a small leucine-rich proteoglycan was particularly up-regulated in double knockdown, we immunostained for decorin in the incisors of 3-month-old F_2_-generation *Cebpb*^−/−^*Runx2*^+/−^ mice (Fig. 6). Positive staining was observed in the enamel epithelial pearl and extracellular matrix near the supernumerary tooth in the periapical region, as well as the extracellular matrix of the cell component surrounding cementum-like hard tissue in the dental pulp. These *in vitro* experiments indicated that knockdown of both *Cebpb* and *Runx2* contributed to EMT in a synergistic manner, whereas *Cebpb* knockdown alone caused down-regulation of *Sox2*.

## Discussion

**Figure 7:**
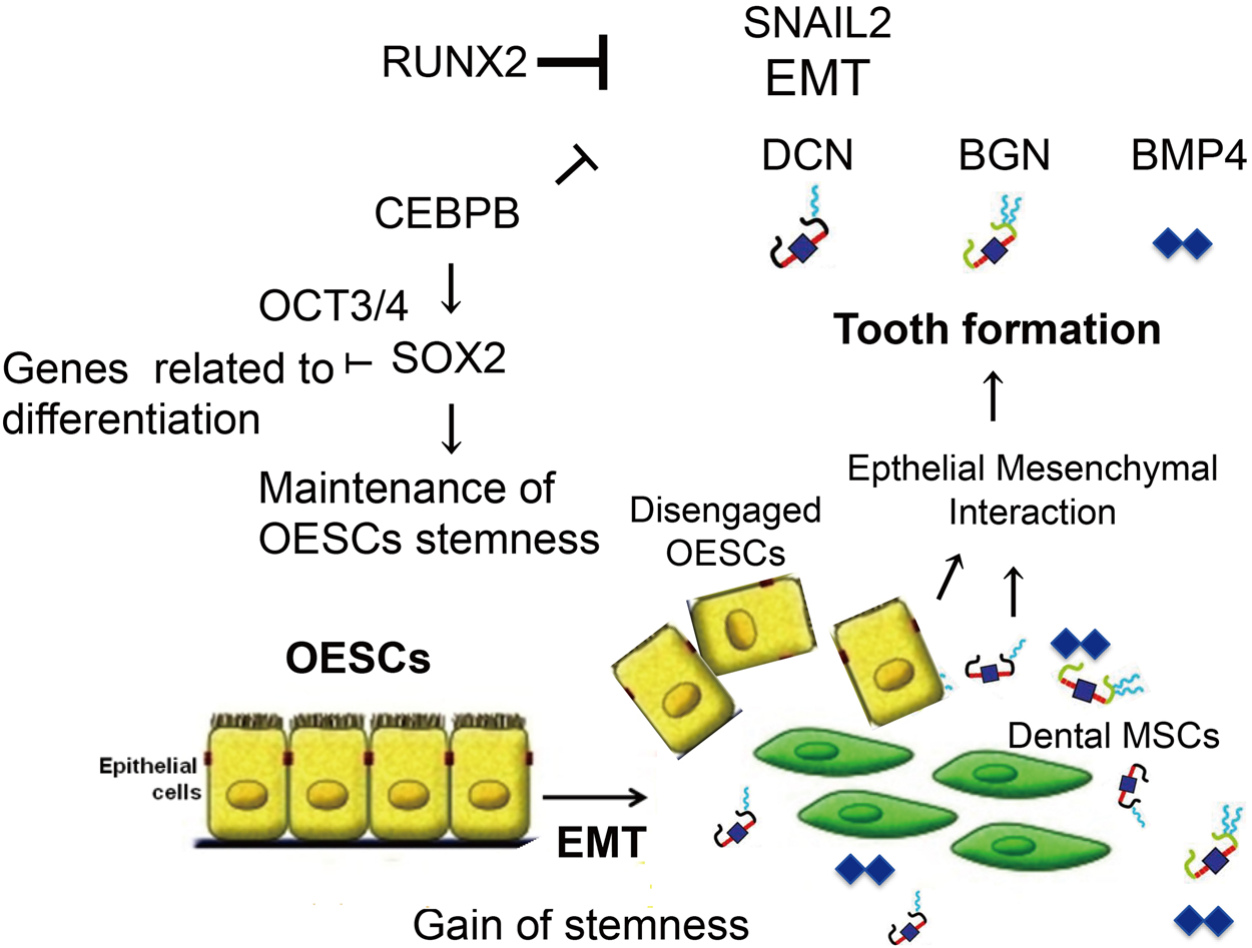
Schematic representation of the role played by *Cebpb* and *Runx2* in odontogenic epithelial stem cells (OESCs). *Cebpb* acts upstream of SRY (sex determining region Y)-box 2 (*Sox2*). In cooperation with *Oct 3/4* protein (*Pou5f1*), Sox2 inhibits genes related to differentiation and maintains stemness in OESCs. *Cebpb* and *Runx2* prevent epithelial-mesenchymal transition (EMT) of OESCs via snail family zinc finger 2 (*Snai2*). Both also inhibit biglycan (*Bgn*), decorin (*Dcn*) and *Bmp4*. EMT promotes expression of *Sox2* and thus stemness of OESCs. EMT enables disengaged OESCs to develop supernumerary teeth by interaction with various mesenchymal stem cells such as dental MSCs.

The *Bmp* signaling network may provide a niche supporting transient Sox2-positive dental epithelial stem cells in murine incisors^31^. In undifferentiated mesenchymal stem cells (MSCs), CEBPB acts as a repressor, helping maintain stemness. Upon adipogenic, chondrogenic, or osteogenic stimulation, different signaling pathways activate Smad proteins, these bind to CEBPB and remove it from its target regulatory elements, thus converting the repressor into an activator^32,33^. Next, CEBPB binds to and activates its differentiation target genes and, together with other lineage-specific transcription factors, promotes mesenchymal differentiation into adipocytes, osteoblasts or chondrocytes. Here, part of the dental pulp caused ectopic cementogenesis in the maxillary incisor of *Cebpb*^−/−^ mice (F_2_ background) (Fig. 3C,H,J and Table 1, Figure Supplement 3A,B). The phenotype was thought to arise from transdifferentiation of OESCs, as these cells invaded the incisor dental pulp rather than its root analogue. Some cementum-producing cells (cementoblasts) may originate from dental epithelial cells^34^. *Cebpb* knockout promotes the differentiation of crown epithelia into the root lineage or even cementoblast-like cells rather than ameloblasts^35^ (Figure Supplement 3C–J). Taken together, these findings suggest that *Cebpb* maintains OESCs in the labial cervical loop epithelium and dental pulp stem cells (DPSCs) in murine incisors (Fig. 7).

In *Runx2* heterozygous and null mice, budding was observed in maxillary incisors at E15 (Fig. 2C,D). Both *Runx2* mutants displayed lingual buds in front of the maxillary molars, which are in line with *Runx2* preventing the formation of buds for successional teeth^24,25^. There is a difference between the phenotypes of OESCs in *Cebpb*^−/−^*Runx2*^+/+^ and *Cebpb*^−/−^*Runx2*^+/−^ incisors (F_2_ background) between E15 and adult animals. No buddings were observed at E15 in *Cebpb*^−/−^ mice (F_2_ background) (Fig. 2B), however they were seen at E15 in *Cebpb*^+/+^*Runx2*^+/−^ and *Cebpb*^−/−^*Runx2*^+/−^ mice (Fig. 2C,D) and disappeared on postnatal day 7 (Figure supplement 4). Meanwhile, some adult *Cebpb*^−/−^ mice (129 Sv background) possessed an unusual incisor that presented ectopic hyperplasia of enamel and dentin in the periapical tissue. Moreover, 33% of 3-month-old *Cebpb*^−/−^*Runx2*^+/−^ mice (F_2_ background) had aberrant incisors, characterized by developing or mature ectopic supernumerary teeth in the periapical tissue and dental pulp (Table 1, Figs 1B–D and 3A–D,H–K). Indeed, in humans, supernumerary teeth are less common in deciduous dentition (first generation of teeth) than in permanent dentition (second generation of teeth)^36^. In mice, the difference may be linked to stem cell aging in the incisor. Common contributing factors of aging in different organisms, but particularly in mammals, are genomic instability, telomere attrition, epigenetic alterations, loss of proteostasis, deregulated nutrient sensing, mitochondrial dysfunction, cellular senescence, stem cell exhaustion, and altered intercellular communication^37^. As another example of epithelial-mesenchymal interactions, hair graying is the most obvious sign of aging in mammals. Irreparable DNA damage, as that caused by ionizing radiation, abolishes renewal of MSCs in mice and results in hair graying inasmuch as it also triggers MSC differentiation into mature melanocytes in the niche^38^. The hallmarks of OESCs can change according to aging.

Differentiated ameloblasts lost their cellular polarity and derangement of ameloblasts was observed in *Cebpb*^+/+^*Runx2*^+/−^, *Cebpb*^−/−^*Runx2*^+/+^, and *Cebpb*^−/−^*Runx2*^+/−^ mice (F_2_ background) (Fig. 4B–E). Furthermore, we observed the positive staining of N-cadherin during EMT within the dental epithelium in the periapical region (Fig. 4F,G). SqRT-PCR analysis of *Runx2* knockdown by siRNA in mHAT9d cells resulted in decreased expression of the epithelial marker E-cadherin (Fig. 5C). The same cells exhibited also increased expression of EMT markers *Cdh2* (N-cadherin) and *Snai2*, but not the other mesenchymal marker *Vim*. It should be noted that *Cebpb* knockdown confirmed increased expression of *Snai2*, as well as a decline in *Vim* (Fig. 5C). This *in vitro* knockdown experiment may be unable to reproduce *in vivo* conditions sufficiently and may only reflect the initial state of EMT, carried out by epithelial cell rests of Malassez (ERM). Nevertheless, these results indicate the additive effect of *Cebpb* and *Runx2* type1 knockdown on EMT. Rodent incisors are a continuation of Hertwig’s epithelial root sheath (HERS), which consists of two epithelial layers and plays a role in inducing odontogenesis during root development, becoming fragmented after that. The fragmentation of HERS cells is associated with EMT^39–41^. Growth inhibition of the inner enamel epithelium and the active proliferation of the outer enamel epithelium and/or stellate reticulum result in HERS formation^42^. ERM are capable of undergoing EMT under osteogenic conditions, when *Vim* is down-regulated^43^, as reported also in this study. Strong *Bmp* signaling in crown/labial dental epithelia acts in synchrony with weak *Wnt* signaling to trigger amelogenesis. The balance between strong *Wnt* signaling and weak *Bmp* signaling in apical/lingual dental epithelia promotes changes in adhesion properties and thus the formation of HERS/ERM. Cessation of *Bmp* signaling and up-regulation of *Wnt* signaling in dental epithelia stimulates the precocious formation of HERS/ERM and epithelium-originated cementogenesis via EMT^34^. *Runx2* is the direct downstream target of *Bmp* signaling^44^. Down-regulation of *Runx2* promotes a similar phenotypic change, as indicated by the onset of EMT in BMPR-1A-depleted tissues during the differentiation stage^35^. *Cebpb* might also be regulated by *Bmp* signaling^45^. Knockout of *Cebpb* mimics a situation characterized by strong *Wnt* signaling and no *Bmp* signaling, thereby leading to EMT and the cementum-like structure of ameloblasts. Therefore, we believe that EMT enabled disengaged OESCs bud and develop supernumerary teeth following induction of DPSCs, MSCs originating from OESCs, or those present in connective tissue near the periapical region. These MSCs may have interacted with free ameloblasts, stimulating their differentiation and the formation of amelogenin-positive enamel epithelial pearls and free blobs. SqRT-PCR analysis of *Runx2* knockdown by siRNA in mHAT9d cells showed that *Sox2* increased during EMT, allowing cells to gain stemness (Fig. 5A). *Cebpb* knockdown and *Runx2* type1 knockdown act on stemness in an opposing manner (Fig. 5A). This behavior has been reported to indicate a direct link between EMT and acquisition of epithelial stem cell properties including self proliferation^46,47^. SqRT-PCR analysis demonstrated increased expression of *Bmp4/6* in *Runx2* type1 knockdown cells and even more so in *Cebpb* and *Runx2* type1 knockdown cells (Fig. 5D). *Bmp* signaling plays a role in tumor generation and progression by affecting tumor EMT and stemness, thus increased *Bmp4/6* expression could induce EMT and increased *Sox2* expression *in vitro*^48^. Taken together, *Runx2* inhibits EMT, differentiation, and branching of undifferentiated enamel epithelial cells, whereas *Cebpb* and *Runx2* knockdown contribute to EMT additively, with EMT promoting *Sox2* mRNA expression and acquisition of stemness (Fig. 7).

Decorin and biglycan are small leucine-rich proteoglycans distributed intracellularly, on cell surfaces, and within extracellular matrices. Biglycan and decorin regulate early stages of tooth formation and mediate mineralization of dental hard tissues. Both *Bgn* and *Dcn* null mice exhibited hypomineralized dentin, with the effect being more conspicuous in the latter. Enamel formation increased strikingly in newborn *Bgn* null mice and was retarded in the case of *Dcn* deletion^49^. The expression of *Bgn* in odontoblasts is highly differentiation stage-dependent. *Dcn* mRNA is expressed by odontoblasts abutting the premineralized matrix and predentin, and is synchronously related to expression of type I collagen. *Bgn* mRNA is expressed by pre-secretory and secretory ameloblasts^50^. *Bgn* may be associated with odontoblast and ameloblast differentiation, enamel formation, and restraining of collagen fibrillogenesis in predentin. Meanwhile, *Dcn* may be committed to dentin formation as a regulator of collagen mineralization, possibly through its specific association with type I collagen fibrils^49–51^. SqRT-PCR analysis demonstrated increased expression of *Bgn*, *Dcn*, and *Bmp4/6* in *Runx2* type1 knockdown cells and even more so in *Cebpb* and *Runx2* type1 knockdown cells (Fig. 5D). From the above results, it appears that increased *Bgn* and *Dcn* expression in OESCs is likely involved in promoting the development of supernumerary teeth in *Runx2*^+/−^*Cebpb*^−/−^ mice. BGN is essential for *Bmp4*-induced osteoblast differentiation in neonatal murine calvarial cells^52^. BGN may drive tooth formation by cooperating with *Bmp4*. DCN inhibits multiple growth factors, such as *Tgfb1*, and directly antagonizes several members of the receptor tyrosine kinase (RTK) family, including the epidermal growth factor receptor, the hepatocyte growth factor receptor, and the insulin-like growth factor receptor I^53^. Soluble decorin has been characterized as a pan-RTK inhibitor, with a simultaneous powerful downstream signaling inhibitory effect^54^. Higher *Dcn* expression enabled the collagen fibers to be markedly thinner and caused no mineralized matrices *in vitro*^55^. These functions of decorin may prevent proliferation of dental epithelial cells and mineralization of mesenchymal cells in the dental pulp, and help the development of a nearly normal supernumerary tooth conformation in the periapical tissue of *Cebpb*^−/−^*Runx2*^+/−^ mice (F_2_ background) (Figs 3C,D and 6, and Table 1).

Molecular targeted therapy uses small molecules, monoclonal antibodies, or other substances to identify and attack specific types of cells by interfering with cellular targets. It could be used for de novo whole tooth regeneration by stimulating arrested tooth germs in animal models with congenital tooth agenesis^16^. Tooth development is under genetic control and is the result of reciprocal and reiterative signaling between oral ectoderm-derived dental epithelium and cranial neural crest cell-derived dental mesenchyme^56^. Two different types of cell components (epithelial and mesenchymal cells) are necessary and essential for whole tooth regeneration. Our results demonstrate that OESCs had the potential to differentiate odontoblasts by EMT and regenerate de novo whole teeth (supernumerary teeth) by synergistic abrogation of *Cebpb* and repression of *Runx2*. In recent years, there have been many studies on stem cells in relation to the development of regenerative therapies for various organs. This has led to the discovery that adult zebrafish regenerate nephrons (the functional units of the kidney) throughout their lives. The zebrafish kidney contains renal stem cells, which differentiate into epithelial or mesenchymal cells of the nephrons^57^. The EMT in *Sox2*-expressing stem/progenitor cells contributes to the regeneration of the pituitary gland^58^. It has been confirmed that *Sox2*, which is one of the molecular marker of OESCs, localizes to the dental lamina of developing human primary molars^59^. OESCs are suitable for molecular targeted therapy using *Cebpb* and/or *Runx2* (Fig. 7). However, from our analysis, it remains unclear whether OESCs differentiate into odontogenic epithelia and mesenchyme directly or through other cells. Future studies tracking individual cells are needed to demonstrate cell dynamics during de novo whole tooth regeneration.

## Materials and Methods

### Production and analysis of *Cebpb* and *Runx2* mutant mice

This study received appropriate approval from the Animal Research Committee of Kyoto University (Reference Number: Med Kyo 11518), as well as from the Recombinant DNA Experiment Safety Committee of Kyoto University to perform recombinant DNA experiments. Experiments were carried out in accordance with approved guidelines.

*Cebpb*^+/−^ and *Runx2*^+/−^ mice were gifts from Shizuo Akira (Osaka University) and Toshihisa Komori (Nagasaki University), respectively^22,60^. *Cebpb*^+/−^ mice were maintained in a 129 Sv background. *Runx2*^+/−^ mice were maintained in a C57BL/6 background. We interbred heterozygous *Cebpb* and *Runx2* mice and analyzed the F_2_ generation. *Runx2* null mice were embryonic lethal. Day E0 was taken to be midnight prior to finding a vaginal plug. Wild-type mice were used as control.

PCR amplification was performed with KOD FX NEO polymerase (KFX-201, TOYOBO, Osaka, Japan) and specific primers.

Three-dimensional (3D) computed tomography (CT) scans (SMX-100XT-SV3; Shimadzu, Kyoto, Japan) were performed to the heads of adult mice. We converted CB to TIFF files. CB files have 512 × 512 pixels, 8 bits, and voxel size x:y:z = 1:1:1 (approx. 0.06 mm per side). 3D images were reconstructed and analyzed using computer imaging software (INTAGE Realia and Volume Player; KGT Inc., Tokyo, Japan)^61^.

### Immunohistochemistry

Heads of adult mice were decalcified with Kalkitox (Wako, Osaka, Japan) overnight at 4 °C and neutralized with 5% sodium sulfate solution (Wako) for more than 2 h. Paraffin-embedded sections were subjected to immunostaining with primary anti-Sox2 rabbit monoclonal antibody (1:100) (ab92494; Abcam, Cambridge, UK), and secondary anti-rabbit antibody (414341; Nichirei Bioscience, Tokyo, Japan), and were counterstained with hematoxylin (Wako). Some sections were immunostained with a primary anti-amelogenin antibody (1:500) (Hokudo, Sapporo, Japan) and a secondary Alexa Fluor 568 goat anti-rabbit antibody (A11036; Thermo Fisher Scientific, Waltham, MA, USA). Subsequently, sections were counterstained with Hoechst 33258 (H3569; Thermo Fisher Scientific). Other sections were immunostained with a primary anti-decorin goat polyclonal antibody (N-15, sc-22613; Santa Cruz Biotechnology, Dallas, TX, USA), and a secondary anti-goat antibody (414351; Nichirei Bioscience); and diaminobenzidine (415171; Nichirei Bioscience). The other sections were immunostained with a primary anti-N-cadherin Rabbit polycloneal antibody (1:100) (ab18203; Abcam) and a EnVision System HRP-labeled polymer (rabbit, K4003; Dako) with diaminobenzidine (K3468; Dako). They were also counterstained with hematoxylin.

### *Cebpb* and *Runx2* siRNA in a mouse enamel epithelial stem cell line

#### Cell culture

mHAT9d is a dental epithelial stem cell line derived from the labial cervical loop epithelium of a mouse incisor. Cells were cultured in Dulbecco’s modified Eagle’s medium/F12 (Thermo Fisher Scientific) containing B-27 supplement (Thermo Fisher Scientific), basic fibroblast growth factor (25 ng/mL; R&D Systems, Minneapolis, MN, USA), epidermal growth factor (100 g/mL; R&D Systems), and penicillin-streptomycin (1%). Cells were cultured on Primaria tissue culture plates (BD Biosciences, Franklin Lakes, NJ, USA). Mycoplasma detection was performed using the PCR Mycoplasma Detection Set (TaKaRa Bio, Shiga, Japan).

#### Examination of transfection efficiency using fluorescent oligos

For each transfection reaction on a 24-well plate, fluorescent oligo-Lipofectamine^®^ RNAiMAX (Thermo Fisher Scientific) complexes were prepared as follows. BLOCK-iT^TM^ Alexa Fluor Red Fluorescent Control (Thermo Fisher Scientific) (6 pmol) was diluted in 100 μL of Opti-MEM^®^ I Reduced Serum Medium (Thermo Fisher Scientific). Lipofectamine^®^ RNAiMAX (0.5, 1.0, and 1.5 μL) was added to the mixture and incubated at room temperature for 15 min. The cell suspension (500 μL) was diluted with the above-mentioned medium and was added to a well containing the fluorescent oligo and Lipofectamine^®^ RNAiMAX mixture. This gave a final fluorescent oligo concentration of 10 nM, a final volume of 600 μL, and 2 × 10^5^ cells. The cells were incubated at 37 °C in a CO_2_ incubator until they were ready for harvesting and assayed for the gene of interest. Transfection efficiency and thus target gene knockdown was measured using a FACS calibur (BD Biosciences) 24, 48, and 72 h after transfection.

#### Gene knockdown using stealth RNAi siRNA in mHAT9d cells

Stealth RNAi siRNA (120 pmol) was diluted in 100 μL of Opti-MEM^®^ I Reduced Serum Medium in a 10-cm dish. Stealth siRNA was as follows: Stealth RNAi^TM^ siRNA Negative Control Med GC Dup, *Cebpb* (NM_001287739.1_stealth_925, Sense: 5′-GAGAGCUCAGCACCCUGCGGAACUU-3′, Anti-Sense: 5′-AAGUUCCGCAGGGUGCUGAGCUCUC-3′), *Runx2* type1 (NM_001145920.2_stealth_1623, Sense: 5′-GGCCACUUACCACAGAGCUAUUAAA-3′, Anti-Sense: 5′-UUUAAUAGCUCUGUGGUAAGUGGCC-3′). Stealth siRNA was mixed with 30 µL Lipofectamine^®^ RNAiMAX and 10 mL of cell suspension diluted with culture medium. This gave a final fluorescent oligo concentration of 10 nM, a final volume of 12 mL, and 4 × 10^6^ cells. mRNA and protein expression at 48 h post transfection were measured using semi-quantitative RT-PCR and western blotting, respectively.

#### SqRT-PCR

RT-PCR was performed as follows. Total RNA from 70% confluent mHAT9d cells was extracted using TRIzol reagent (Thermo Fisher Scientific), 0.2 mL chloroform, and 0.2 mL phenol chloroform per 1 mL of TRIZOL. The RNA was precipitated from the aqueous phase by mixing with isopropyl alcohol and 16.7 mg/mL glycogen or with NucleoSpin^®^ RNA Clean-up XS columns (U0903, TaKaRa Bio). Total RNA (3 μg) was reverse transcribed using the SuperScript ^®^IV First-Strand Synthesis System (Thermo Fisher Scientific).

cDNA was serially diluted and PCR amplification was performed using Ex Taq (RR001; TaKaRa Bio), KOD FX (KFX-101; TOYOBO), and specific oligonucleotide primers (Supplementary Table 1, 2). All PCR products were examined with serial dilutions of the cDNA by electrophoresis on 2% agarose gels with ethidium bromide staining. The resulting bands were quantified with a Bio-image analyzer (FAS-IV; NIPPON Genetics, Tokyo, Japan). Experiments were carried out in triplicate. To characterize the mHAT9d cell line, we used RT-PCR analysis to confirm the expression of dental epithelial cell markers: cytokeratin 14 (*Krt14*), notch 1 (*Notch1*), jagged 1 (*Jag1*), and p63 (*Tprg*); core binding factor beta 1 (*Cbfb1*),Polycomb complex protein BMI-1 (*Bmi1*), ras homolog family member A (*Rhoa*), and fibronectin (*Fn1*); small integrin-binding ligands of the N-linked glycoprotein family members dentin matrix protein1 (*Dmp1*) and osteopontin (*Spp1*); and the neural crest marker proteolipid protein 1 (*Plp1*)^62^; *P-cadherin* (*Cdh3*)*;* transforming growth factor beta (*Tgfb*)*1/2/3*; the *Bmp* antagonist-sclerostin domain containing 1 (*Sostdc1*) (Supplemental Fig. 5, Supplementary Table 2).

Each siRNA was the same in quantity (10 nM each) when mixed. 1/10, 1/30, and 1/90 indicates dilution ratio. 925 and 1623 indicate *Cebpb* and *Runx2* type1 siRNA, respectively. RT-PCR analysis of *Cebpb and Runx2* type1 expression after knockdown with negative control stealth siRNA or the corresponding siRNA (final concentration 20 nM). All experiments were performed at least thrice, and representive data are shown. GAPDH was used as an internal control for RT-PCR. The bar graphs indicate relative mRNA level of genes, which were calculated by image j software. Values of them were normalized to GAPDH and mRNA expression data on negative control stealth siRNA were used as denominator of ratios.

#### Western blotting

Western blotting of mHAT9d cells transfected with *Cebpb* and *Runx2* type1 stealth siRNA was performed using the following antibodies: anti-Gapdh primary rabbit polyclonal (1:1000, sc-150; Santa Cruz Biotechnology), secondary anti-rabbit IgG (1:5000, #7074; Cell Signaling Technology), anti-C/EBP beta primary rabbit polyclonal (1:200, #3087; Cell Signaling Technology), anti-Runx2 primary rabbit polyclonal (1:200, M-70 and sc-10758; Santa Cruz Biotechnology), and secondary anti-rabbit IgG (1:1000, #7074; Cell Signaling Technology). Ez West Blue (AE-1490; ATTO, Tokyo, Japan) was used to stain the membranes and detect the proteins of interest.

### Statistical analysis

Data are presented as mean ± standard deviation. Statistical significance was assessed by analysis of variance in R. Gaussian distribution was determined with an Anderson-Darling normality test.

To analyze the labial cervical loop epithelium area, the number of Sox2-positive cells and Sox2-positive area/Labial cervical loop epithelium area, which was measured using the imageJ software, in the maximal area of all sections in *Cebpb* genetically modified mice (129 Sv), statistical significance was determined using a Mann-Whitney U test. Sox2-positive area (diaminobenzidine coloring) was extracted with Photoshop elements 12 (Adobe Systems, San Jose, CA, USA). To analyze the area and the cell number of lingual buds in the maximal area of all sections in *Cebpb*^+/+^*Runx2*^+/−^ and *Cebpb*^−/−^*Runx2*^+/−^ F_2_ (129 Sv/C57BL/6) mice, statistical significance was determined using a Mann-Whitney U test.

## Electronic supplementary material

Supplemental information
