# Supplementary material for: Loss of Stemness, EMT, and Supernumerary Tooth Formation in Cebpb−/−Runx2+/− Murine Incisors
Source: Sci Rep. 2018 Mar 26;8:5169. doi: 10.1038/s41598-018-23515-y (PMC5980103; doi:10.1038/s41598-018-23515-y)
Supplement: Supplementary file 1 — Supplemental information [file 41598_2018_23515_MOESM1_ESM.docx]

**Supplementary information**

**Loss of Stemness, EMT and Supernumerary Tooth Formation in *Cebpb*^-/-^*Runx2*^+/-^ Murine Incisors**

Kazuyuki Saito^1,5^, Katsu Takahashi^1,✴^, Boyen Huang^2^, Masakazu Asahara^3^, Honoka Kiso^1^, Yumiko Togo^1^, Hiroko Tsukamoto^1^, Sayaka Mishima^1^, Masaki Nagata^4^, Machiko Iida^5^, Yoshihito Tokita^5^, Masato Asai^5^, Akira Shimizu^6^, Toshihisa Komori^7^, Hidemitsu Harada^8^, Mary MacDougall^9^, Manabu Sugai^10,✴^, Kazuhisa Bessho^1^

^1^Department of Oral and Maxillofacial Surgery, Graduate School of Medicine, Kyoto University, Kyoto, Japan

^2^Professor of Dentistry and Head of School, School of Dentistry and Health Sciences, Faculty of Science, Charles Sturt University, Leeds Parade Orange NSW 2800 Australia

^3^Division of Liberal Arts and Sciences, Aichi Gakuin University, Aichi, Japan

Department of Oral and

Maxillofacial Surgery, Niigata University Graduate School of Medical

and Dental Sciences, 2-5274 Gakkocho-dori, Niigata 951-8514, Japan

Department of Oral and

Maxillofacial Surgery, Niigata University Graduate School of Medical

and Dental Sciences, 2-5274 Gakkocho-dori, Niigata 951-8514, Japan

^4^Department of Oral and Maxillofacial Surgery Niigata University Graduate School of Medical and Dental Sciences, Niigata, Japan

^5^Department of Perinatology, Institute for Developmental Research, Aichi Human Service Center, Kasugai, Aichi, Japan

^6^Department of Experimental Therapeutics, Institute for Advancement of Clinical and Translational Science, Kyoto University Hospital, Kyoto, Japan

^7^Department of Cell Biology, Unit of Basic Medical Sciences, Nagasaki University Graduate School of Biomedical Sciences, Nagasaki, Japan

^8^The Advanced Oral Health Science Research Center, Iwate Medical University, Iwate, Japan.

^9^Institute of Oral Health Research & Department of Oral and Maxillofacial Surgery, School of Dentistry, University of Alabama, Birmingham, Alabama, United States of America

^10^Department of Molecular Genetics, Division of Medicine, Faculty of Medical Sciences, University of Fukui, Fukui, Japan

**^✳^Corresponding author**:

Dr. Manabu Sugai, Department of Molecular Genetics, Division of Medicine, Faculty of Medical Sciences, University of Fukui, Fukui, Japan. Tel.: +81-0776-61-8312, Fax: +81-0776-61-8164, E-mail: [msugai@u-fukui.ac.jp](mailto:msugai@u-fukui.ac.jp)

Dr. Katsu Takahashi, Department of Oral and Maxillofacial Surgery, Graduate School of Medicine, Kyoto University

Shogoin-Kawahara-cho 54, Sakyo-ku, Kyoto 606-8507, Japan. Tel.: +81-75-751-3403, Fax: +81-75-761-9732, E-mail: takahask@kuhp.kyoto-u.ac.jp

**Supplementary information**

The PDF file includes:

Supplementary Figure 1, 2, 3, 4, 5, 6 and table 1, 2


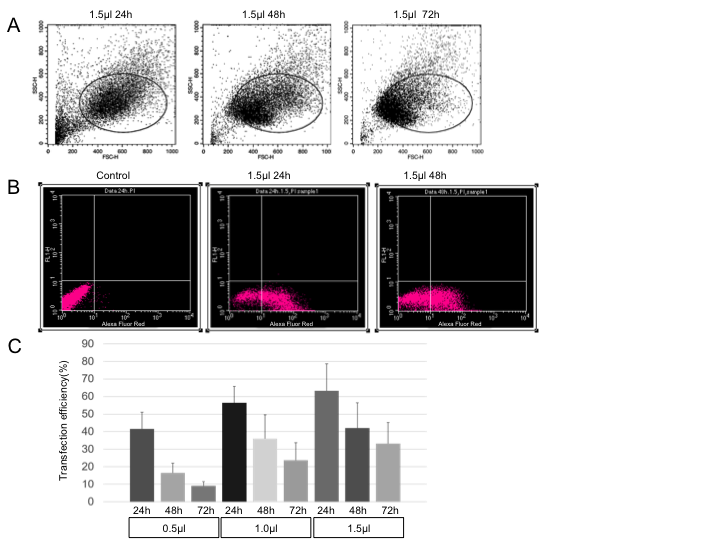


**Supplementary Figure 1 | Transfection efficiency of mHAT9d cells with stealth siRNA.**

Efficiency was measured by fluorescence-activated cell sorting (FACS caliber; BD, NJ, USA) 24, 48, and 72 h after transfection of BLOCK-iT (TM) Alexa Fluor (R) Red Fluorescent Control (Thermo Fisher Scientific, Waltham, MA, USA).

A: Gated dot plots generated by Cell Quest^TM^ Pro Software (BD) indicate forward scatter (X-axis) and side scatter (Y-axis) following transfection with 1.5 μL Lipofectamine^®^ RNAiMAX (Thermo Fisher Scientific).

B: Comparison between control (without transfection), 24h and 48h after transfection with 1.5 μL Lipofectamine^®^ RNAiMAX. Gated dot plots generated by Cell Quest^TM^ Pro Software indicate Alexa Fluor Red (X-axis) and FL-1H (Y-axis)).

C: Efficiency is represented as mean ± standard deviation following transfection with 0.5, 1.0, and 1.5 μL Lipofectamine^®^ RNAiMAX. Number of samples is 4.


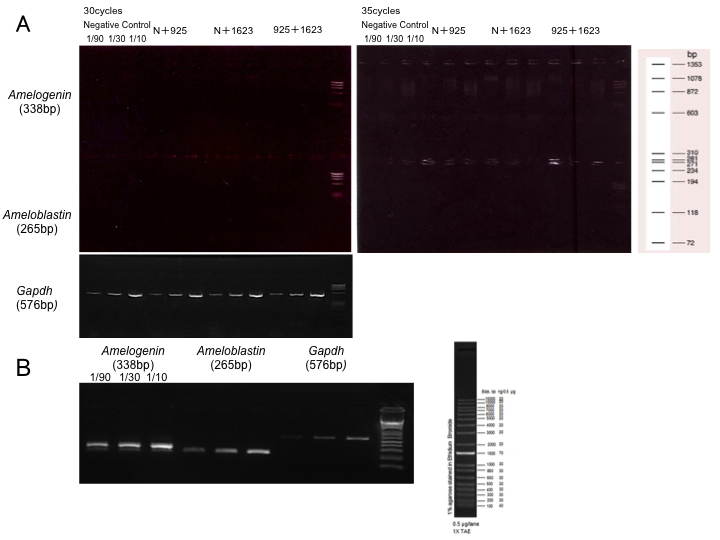


**Supplementary Figure 2 | Semi-quantitative reverse transcription polymerase chain reaction (RT-PCR) of mHAT9d cells transfected with stealth siRNA for amelogenin (*Amelx*) and ameloblastin (*Ambn*).**

A: Total RNA (3μg) was extracted from 70% confluent mHAT9d cells and reverse transcribed using a SuperScript ®IV First-Strand Synthesis System (Thermo Fisher Scientific, Waltham, Massachusetts, USA). The cDNAs were serially diluted and PCR amplification was performed using KOD FX (KFX-101, TOYOBO, Osaka, Japan) and specific oligonucleotide primers for *Amelx,* *Ambn* and glyceraldehyde 3-phosphate dehydrogenase (*Gapdh*) (Supplemental Table 1). No PCR products were detected in 30-35 cycles using a 2% agarose gel electrophoresis with φ×174-HaeⅢdigest (3405A, TaKaRa Bio, Shiga, Japan). N indicates negative control stealth siRNA; 925 and 1623 indicate *Cebpb* and *Runx2* type1 siRNA, respectively (final concentration 20 nM). Each siRNA was the same in quantity (10 nM each) when mixed.

B: Total RNA (0.5µg) was extracted from labial cervical loop epitheliums in apical end of four bimaxillary incisors of a 129sv wild-type mouse and reverse transcribed as stated above. The cDNAs were serially diluted and PCR amplification was performed using KOD FX and specific oligonucleotide primers for *Amelx*, *Ambn*, and *Gapdh*. The PCR products were detected in 25 cycles using a 2% agarose gel electrophoresis with 1kb plus DNA Ladder (10787-018, ThermoFisher scientific).

**
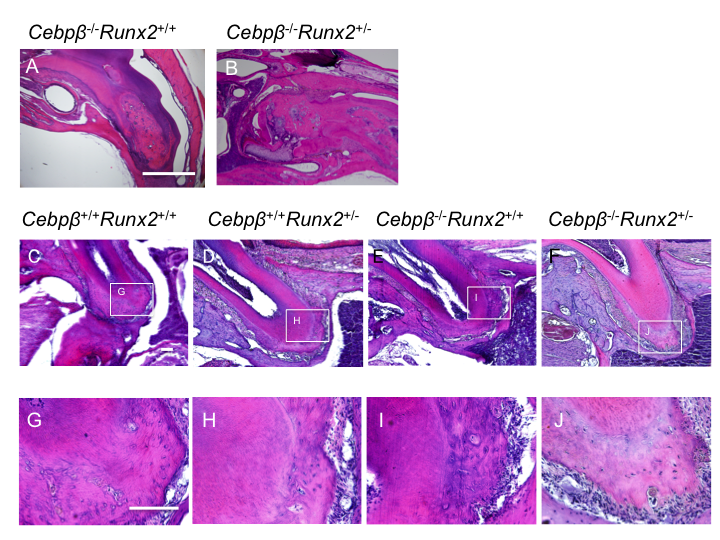
**

**Supplementary Figure 3 | Hematoxylin-eosin (H&E) staining of cementum-like hard tissue in the dental pulp and cementum in adult maxillary molars in *Cebpβ* *and/or Runx2* double genetically modified mice (F_2_: 129Sv/C57BL/6) at 3 months after birth.**

A, B: Cementum-like hard tissue of dental pulp in maxillary incisors in *Cebpb*^-/-^*Runx2*^+/+^ or *Cebpb*^-/-^*Runx2*^+/-^ mice. Scale bar: 1mm, 40×.

C-F: Root with cementum in mouse molar (M1)×100 Scale bar: 100 μm, 100×.

G-J: Higher magnification image of cellular intrinsic fiber cementum (CIFC). Scale bar: 100 μm, 400×.

C, D, G, H: Margin of CIFC is smooth shaped in wild-type or *Runx2* heterozygnous mice.

E, F, I, J: Margin of CIFC is invasive type and rough-edged in *Cebpb* null mice.

**
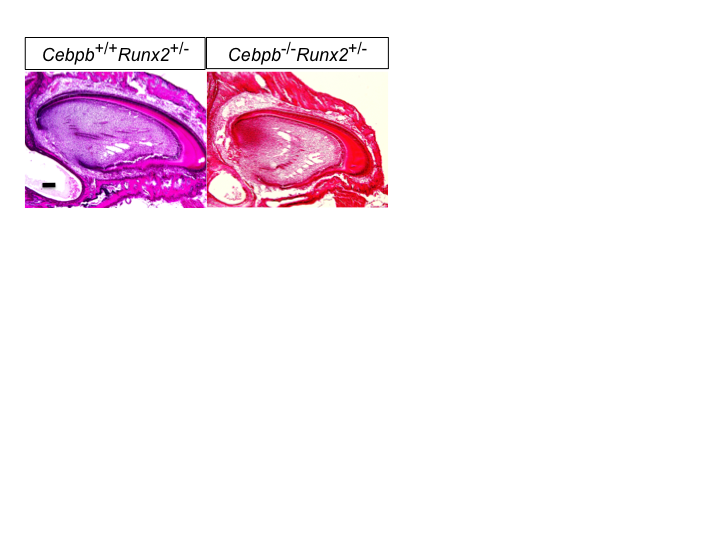
**

**Supplementary Figure 4 |** **the upper incisors of *Cebpb*^+/+^*Runx2*^+/-^ and *Cebpb*^-/-^*Runx2*^+/-^ F_2_ mice (129Sv/C57BL/6) on day 7 after birth.**

No supernumerary tooth formation of the incisors of both mice. Scale bar: 100μm.

**
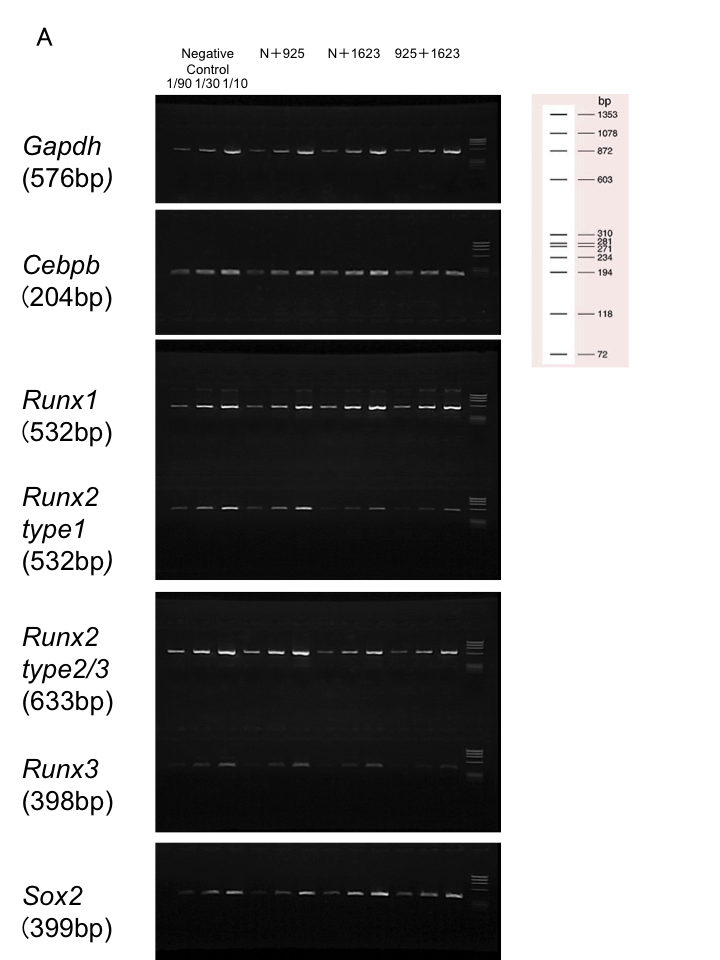

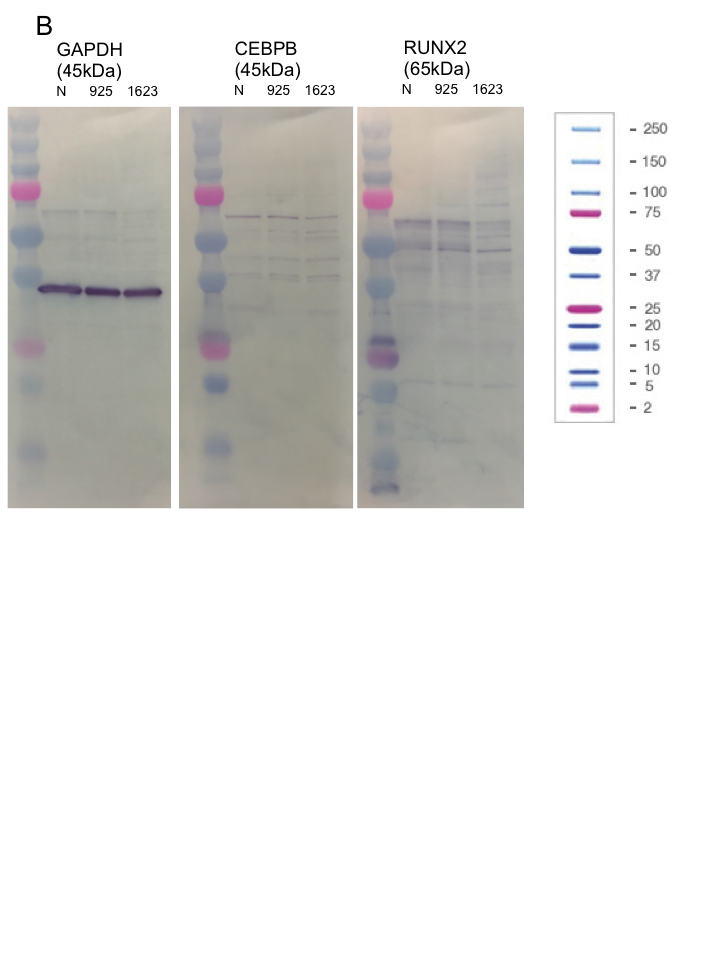

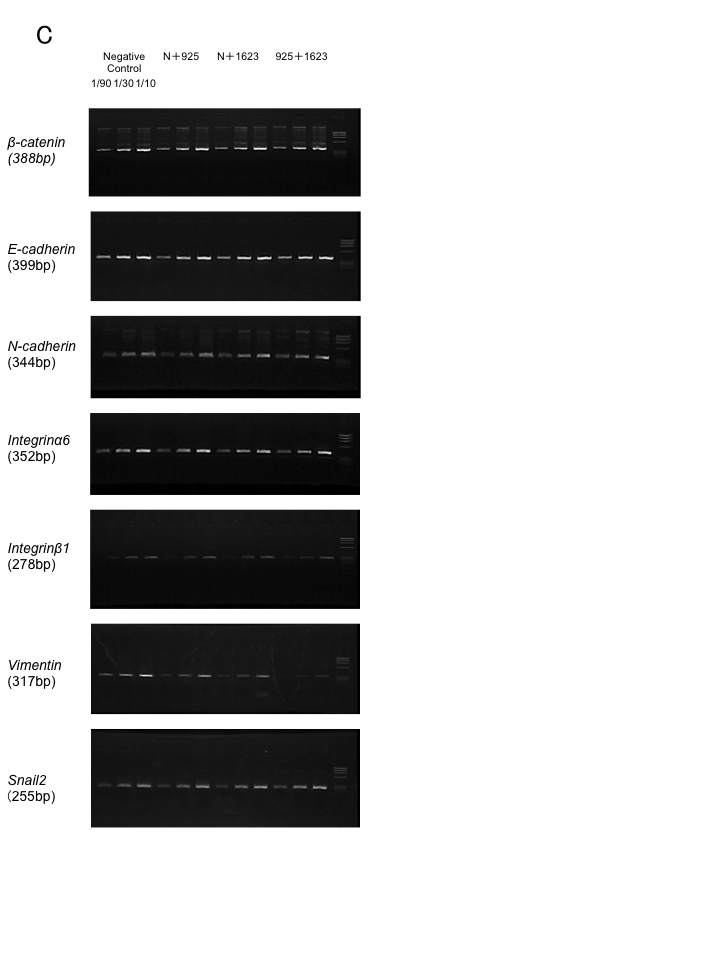
**

**
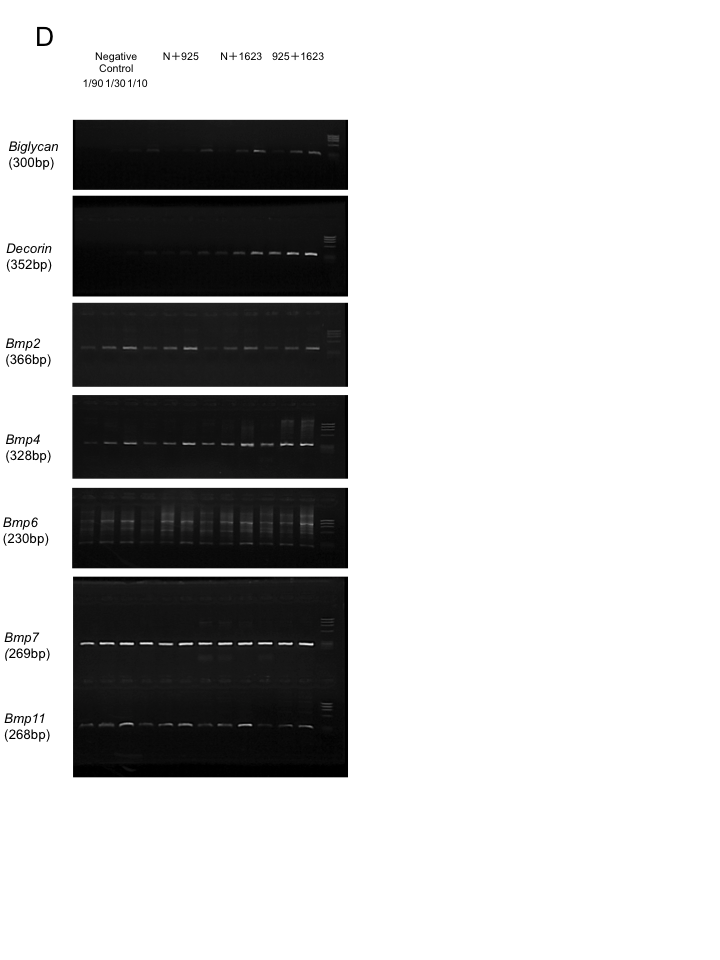
**

**Supplementary Figure 5 |Full-length gels and blots in Figure 5.**

A, C, D: Total RNA (3μg) was extracted from 70% confluent mHAT9d cells and reverse transcribed using a SuperScript ®IV First-Strand Synthesis System (Thermo Fisher Scientific, Waltham, Massachusetts, USA). The cDNAs were serially diluted and PCR amplification was performed using KOD FX (KFX-101, TOYOBO, Osaka, Japan) and specific oligonucleotide primers and glyceraldehyde 3-phosphate dehydrogenase (*Gapdh*) (Supplemental Table 1). PCR products were detected using a 2% agarose gel electrophoresis with φ×174-HaeⅢdigest (3405A, TaKaRa Bio, Shiga, Japan). N indicates negative control stealth siRNA; 925 and 1623 indicate *Cebpb* and *Runx2* type1 siRNA, respectively (final concentration 20 nM). Each siRNA was the same in quantity (10 nM each) when mixed. B: Western blotting results.Western blotting of mHAT9d cells transfected with *Cebpb* and *Runx2* type1 stealth siRNA was performed using the following antibodies: anti-*Gapdh* primary rabbit polyclonal (1:1000, sc-150; Santa Cruz Biotechnology), secondary anti-rabbit IgG (1:5000, #7074; Cell Signaling Technology), anti-C/EBP beta primary rabbit polyclonal (1:200, #3087; Cell Signaling Technology), anti-*Runx2* primary rabbit polyclonal (1:200, M-70 and sc-10758; Santa Cruz Biotechnology), and secondary anti-rabbit IgG (1:1000, #7074; Cell Signaling Technology). Ez West Blue (AE-1490; ATTO, Tokyo, Japan) was used to stain the membranes and detect the proteins of interest.

**
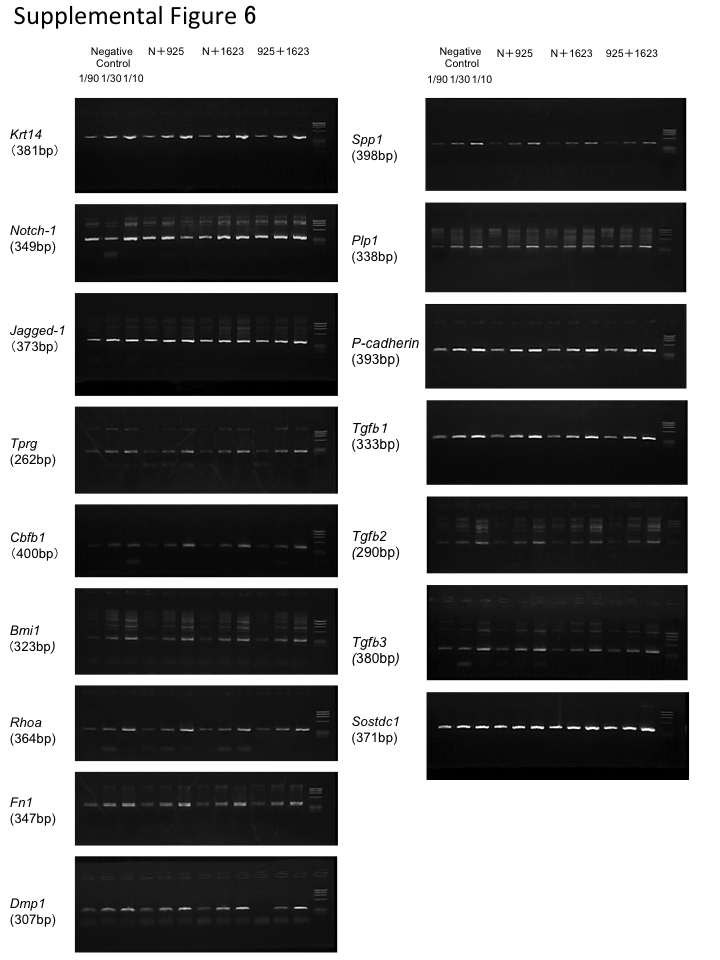

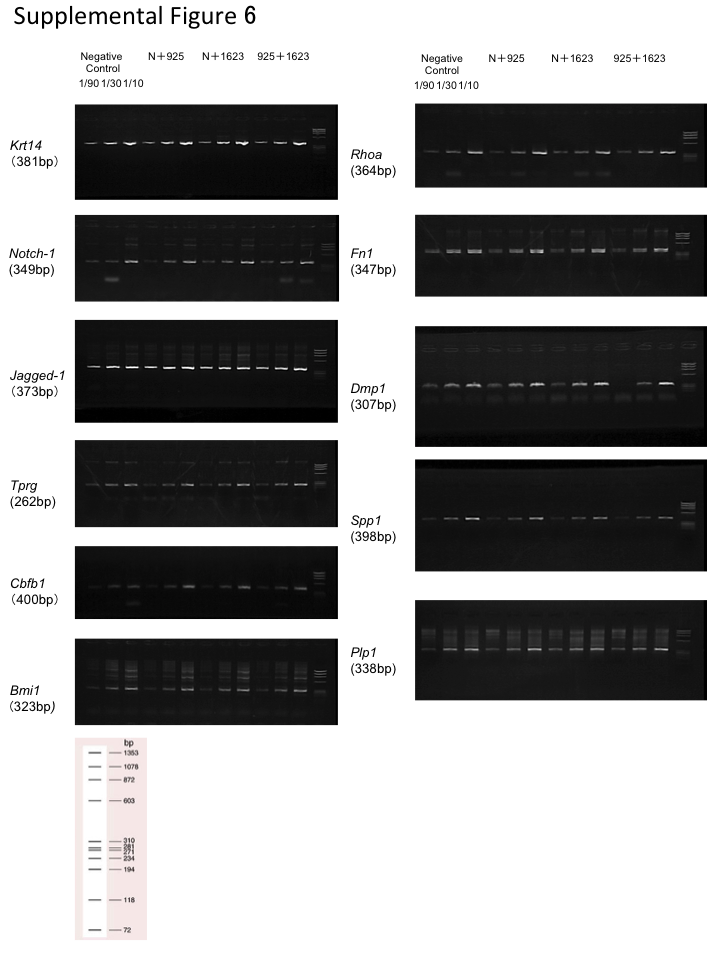
**

**Supplementary Figure 6 | Semi-quantitative reverse transcription polymerase chain reaction (RT-PCR) of mHAT9d cells transfected with stealth siRNA for genes characteristic of them.**

Total RNA was extracted from 70% confluent mHAT9d cells and reverse transcribed using a SuperScript ®IV First-Strand Synthesis System (Thermo Fisher Scientific, Waltham, Massachusetts, USA). The cDNAs were serially diluted and PCR amplification was performed using KOD FX (KFX-101, TOYOBO, Osaka, Japan) and specific oligonucleotide primers (Supplemental Table 2), and glyceraldehyde 3-phosphate dehydrogenase (*Gapdh*) (Supplemental Table 1). PCR products were detected using a 2% agarose gel electrophoresis with φ×174-HaeⅢdigest (3405A, TaKaRa Bio, Shiga, Japan). N indicates negative control stealth siRNA; 925 and 1623 indicate *Cebpb* and *Runx2* type1 siRNA, respectively (final concentration 20 nM). Each siRNA was the same in quantity (10nM each) when mixed.

| Gene name | Forward sequence | Reverse sequence | NCBI accession number |
| --- | --- | --- | --- |
| *Gapdh* | CCATCACCATCTTCCAGGAG | CCTGCTTCACCACCTTCTTG | NM_008084.3 |
| *Cebpb* | ACACGTGTAACTGTCAGCCG | GCTCGAAACGGAAAAGGTTC | NM_001287738.1 |
| *Runx1* | AGAAGTGTAAGCCCAGCACA | TTCTCAGTTCTGCCGAGTAG | NM_001111021.2 |
| *Runx2type2/3* | GAGGGCACAAGTTCTATCTG | CGCTCCGGCCCACAAATCTC | NM_001146038.2 XM_006523544.2 |
| *Runx2type1* | CACTTCGCTAACTTGTGGCTGT | TTCATAACAGCGGAGGCATTT | NM_001145920.2 |
| *Runx3* | GGCTTCCAACAGCATCTTTG | CGGAGTAGTTCTCATCATTG | NM_019732.2 |
| *Sox2* | AACCACCAATCCCATCCAA | CCAGCAAGAACCCTTTCCTC | NM_011443.4 |
| *β-catenin* | TGCTGGTGACAGGGAAGACA | CCGAGCAAGGATGTGGAGAG | NM_007614.3 |
| *E-cadherin* | CCTGTCTTCAACCCAAGCAC | GATTTCCTGACCCACACCAA | NM_009864.2 |
| *N-cadherin* | CGCCTATGAGTGGGACAGGA | ACGCAGGATGGAAATGTTGG | NM_007664.5 |
| *Integrin α6* | CATCCTCCTGGCTGTTCTTG | GGGGCTTTGGGTAGTGTGAG | NM_008397.4 |
| *Integrin β1* | TTAGCACAACCCCAGCAAAG | CCCATCTCCAGCAAAGTGAA | NM_010578.2 |
| *Vimentin* | CAAGAACACCCGCACCAAC | TGCTTTCGGCTTCCTCTCTC | NM_011701.4 |
| *Snai2* | CCTCCAAGAAGCCCAACTACA | TGGATGAAGTGTCAGAGGAAGG | NM_011415.2 |
| *Biglycan* | CCCTGAGACCCTGAACGAAC | ACTCCGAAGCCCATAGGACA | NM_007542.5 |
| *Decorin* | TGCCTGGGCTGCATAGTTAG | GGTTGTGTCGGGTGGAAAA | NM_001190451.2 |
| *Bmp2* | CTGTCTTCTAGTGTTGCTGCTTCC | GCCGTTTTCCCACTCATCTC | NM_007553.3 |
| *Bmp4* | AGGAGGAGGAGGAAGAGCAGA | TCCAGTAGTCGTGTGATGAGGTG | NM_007554.3 |
| *Bmp6* | TCCCACTCAACGCACACA | CACCCACACACACACACCA | NM_007556.3 |
| *Bmp7* | GAGGGCTGGTTGGTGTTTG | TGGTTCTTTGGCGTCTTGG | NM_007557.3 |
| *Bmp11* | AAGTCGCAGATCCTGAGCAAA | TGGGGCTGAAGTGGAAATG | NM_010272.2 |
| *Ameloblastin* | GCCTGATCCTGTTCCTGTCC | GTTTCATGTTCCCTTGGTCCTATC | NM_009666 |
| *Amelogenin* | TTTGTTTGCCTGCCTCCTG | GCTGATGGTGTTGGGTTGG | NM_009664.2 |

**Supplementary Table 1| Specific oligonucleotide primers for reverse transcription polymerase chain reaction (RT-PCR) regarding genes presenting changes of mRNA expression in mHAT9d cells 48 h after transfection with *Cebpb* and *Runx2* type1 stealth siRNA.**

| Gene name | Forward sequence | Reverse sequence | NCBI accession number |
| --- | --- | --- | --- |
| *Krt 14* | GCGAGATGGAGCAGCAGAA | GGACAAGGGTCAAGTAAAGAGTGAA | NM_001313956 |
| *Notch1* | CGCAAGCACCCAATCAAG | CACAGCCCACAAAGAACAGG | NM_008714 |
| *Jag1* | GCTGGATGGGTCCTGATTG | TTATGGCAGGGGTCAGAGAGA | NM_013822 |
| *p63* | TTCCCTATGCCACCTTCACA | CAAAAGCCAATACTCCCACGA | NM_175165 |
| *Cbfb1* | CAGGAACCAATCTGTCTCTCCA | AAGCTGTGCTCCACTTAACGAA | NM_022309 |
| *Bmi1* | TACGATGCCCAGCAGCAA | ACAGGAAGAGGTGGAGGGAAC | NM_007552 |
| *Rhoa* | AATGACGAGCACACGAGACG | CGTGGTTGGCTTCTAAATACTGG | NM_001313961 |
| *Fn1* | CGGGAATGGAAAGGGAGAA | TAGGGTGGGGCTGGAAAGA | NM_010233 |
| *Dmp1* | TGTCATTCTCCTTGTGTTCCTTTG | ACTGTCGTCTTCATCATCCTCCTT | NM_016779 |
| *Spp1* | GATTTGCTTTTGCCTGTTTGG | CTGTAGGGACGATTGGAGTGAA | NM_001204233 |
| *Plp1* | AGCAAAGTCAGCCGCAAAA | TAGAAGCCCTCAGCCAGCA | NM_011123 |
| *P-cadherin* | TTTCCAGGCCCAGCTAACAC | TCACCACCACCCTCTTCTCC | NM_001037809.5 |
| *Tgfb1* | TGACAGCAAAGATAACAAACTCCAC | TACTGTGTGTCCAGGCTCCAA | NM_011577.2 |
| *Tgfb2* | AACCAGAGCGGAGGGTGAA | AGGGCAACAACATTAGCAGGAG | NM_009367.4 |
| *Tgfb3* | CGCAGACACAACCCATAGCA | ACCAACCCACACTTTCTTTACCAC | NM_009368.3 |
| *Sostdc1* | TGGAGGCAGGCATTTCAGTAG | AGTTGTGGCTGGACTCGTTG | NM_025312.3 |

**Supplementary Table 2 | Specific oligonucleotide primers for reverse transcription polymerase chain reaction (RT-PCR) regarding characteristic genes of mHAT9d.**
